# A Novel Function of Tubulin β2a and Tubulin β3 in Regulating Metastatic Properties in Bone-Metastatic Breast Cancer Cells

**DOI:** 10.3390/cancers18213418

**Published:** 2026-10-22

**Authors:** Shreya Patel, Marcus Winogradzki, Kayla M. Gant, Danielle Hazime, Sai Srujana Arra, Niyati Patel, Alan T. Blank, Jitesh Pratap

**Affiliations:** 1 Caris Life Sciences, Irving, TX 75039, USA; 2 Department of Anatomy and Cell Biology, Rush University Medical Center, Chicago, IL 60612, USA; marcus_c_winogradzki@rush.edu (M.W.); kayla_m_gant@rush.edu (K.M.G.); saisrujana_arra@rush.edu (S.S.A.); niyati_x_patel@rush.edu (N.P.); 3 Department of Orthopedic Surgery, Rush University Medical Center, Chicago, IL 60612, USA; alan_blank@rush.edu

**Keywords:** bone metastasis, breast cancer, microtubules, tubulin isotypes, metastatic properties

## Abstract

**Background/Objectives**: Bone metastasis (BM) occurs in approximately 70% of patients with advanced breast cancer (BCa) and is associated with limited treatment options and a median survival of only 19–25 months. Although microtubule (MT)-targeting agents such as paclitaxel remain a standard therapy, their efficacy is often limited by chemoresistance linked to in part by altered β-tubulin isotype expression. Among these, tubulin β2a and tubulin β3 (encoded by *TUBB2A* and *TUBB3*) have been linked to poor clinical outcomes; however, their roles in breast cancer bone metastasis (BMBC) remain unclear. Here, we investigated the functional significance of *TUBB2A* and *TUBB3* in regulating MT dynamics and metastatic behavior in bone-derived BCa cells. **Methods**: *TUBB2A* and *TUBB3* expression levels were evaluated in primary breast tumors and bone metastases. CRISPR-mediated TUBB2A knockout and TUBB3 depletion were performed in bone-derived MDA-MB-231 cells to assess microtubule dynamics, trafficking, metastatic phenotypes, and gene expression. **Results**: Elevated expression of both *TUBB2A* and *TUBB3* correlated with reduced patient survival. Immunohistochemical analyses demonstrated increased tubulin β2a abundance in BM lesions relative to matched primary breast tumors, while the proportion of moderate-to-strong tubulin-β3-positive cells was also increased in BM. Using CRISPR/Cas9-mediated knockout/knockdown in a bone-derived MDA-MB-231 cell model, we found that loss of *TUBB2A* or partial depletion of *TUBB3* impaired MT regrowth, altered the cell cycle, and disrupted endosomal trafficking, characterized by enlarged perinuclear Rab5-positive vesicles. Functionally, depletion of either isotype reduced cell migration and invasion while enhancing adhesion, accompanied by increased expression of integrin β4, β5, α4, α5, and talin-1. Exploratory RNA-seq analysis suggests enrichment of cytokine–cytokine receptor signaling pathways, consistent with a potential link between MT remodeling and inflammatory/secretory programs. **Conclusions**: Collectively, these findings identify *TUBB2A* and *TUBB3* as regulators of metastatic phenotypes and potential biomarkers and therapeutic targets in BMBC.

## Simple Summary

Bone metastasis occurs in approximately 70% of patients with advanced breast cancer and is associated with limited treatment options. While microtubule-targeting drugs such as paclitaxel remain standard therapies, their effectiveness is often reduced by treatment resistance linked to changes in β-tubulin isotype expression. Among these isotypes, *TUBB2A* and *TUBB3* have been associated with poor clinical outcomes, but their roles in breast cancer bone metastasis remain unclear. Here, we show that elevated tubulin *TUBB2A* and *TUBB3* expression is associated with poor patient survival and increased abundance of tubulin β2 and β3 in bone-metastatic lesions. CRISPR-mediated reduction in *TUBB2A* or *TUBB3* impaired microtubule regrowth, disrupted cell-cycle progression and endosomal trafficking, reduced migration and invasion, and increased cell adhesion. Transcriptomic analyses further suggested alterations in inflammatory and secretory signaling pathways. Collectively, our findings identify *TUBB2A* and *TUBB3* as key regulators for metastatic phenotypes and potential therapeutic targets in bone-metastatic breast cancer.

## 1. Introduction

Bone metastases develop in approximately 65–75% of patients with advanced breast cancer (BCa) and represent a major contributor to cancer-associated morbidity and mortality [1]. Despite significant advances in systemic therapies, bone-metastatic breast cancer remains largely incurable. Identifying the molecular mechanisms that enable tumor cells to adapt and thrive within the bone niche is critical for developing more effective therapeutic strategies.

Microtubules (MTs), dynamic cytoskeletal polymers composed of α- and β-tubulin heterodimers, coordinate essential cellular processes implicated in cancer progression, including mitosis, intracellular trafficking, migration, and cell adhesion [2]. Dysregulated MT dynamics are a hallmark of aggressive malignancies and contribute to enhanced invasiveness, metastatic competence, and resistance to chemotherapy [3,4]. Increasing evidence supports the concept of a “tubulin code,” whereby distinct β-tubulin isotypes, encoded by separate genes and defined by unique C-terminal sequences and post-translational modifications, confer functional heterogeneity to the MT network through differential interactions with microtubule-associated proteins (MAPs) and motor proteins [5,6,7].

Among β-tubulin isotypes, tubulin β2a and tubulin β3—encoded by *TUBB2A* and *TUBB3*—have emerged as clinically relevant regulators of cancer behavior. *TUBB3* is frequently overexpressed in aggressive solid tumors and is strongly associated with resistance to microtubule-targeting agents such as taxanes and vinca alkaloids, as well as poor clinical outcomes [8,9,10,11]. TUBB2A has been linked to transforming growth factor-β (TGF-β) signaling and epithelial-to-mesenchymal transition (EMT), a critical program underlying tumor cell invasion and dissemination [12,13]. Despite these associations, the mechanistic roles of *TUBB2A* and *TUBB3* in breast cancer bone metastasis remain poorly defined.

In the context of bone metastasis, MT stability is particularly important for the trafficking and secretion of matrix-modifying enzymes, cytokines, and vesicular cargo that remodel the bone microenvironment. For example, α-tubulin acetylation enhances MT stability and facilitates secretion of pro-osteolytic factors, including RANKL, thereby exacerbating osteolysis [5,14,15]. Alterations in β-tubulin isotype composition may enable metastatic breast cancer cells to adapt to the bone niche by modulating vesicular transport, cytokine release, and interactions with osteoblasts and osteoclasts. However, the contribution of specific β-tubulin isotypes to these processes has not been systematically investigated.

In this study, we found that overall tubulin-β2a-positive tumor cells and the frequency of moderate-to-strong tubulin-β3-positive tumor cells were enriched in bone-metastatic lesions compared with matched primary breast tumors. Furthermore, high expression of either isotype was associated with reduced overall survival, implicating *TUBB2A* and *TUBB3* as potential regulators of metastatic progression and adverse clinical outcomes.

Using CRISPR/Cas9-mediated *TUBB2A* knockout and partial *TUBB3* knockdown in bone-tropic MDA-MB-231 cells, we defined the roles of these isotypes in metastatic breast cancer. Loss of *TUBB2A* and partial depletion of *TUBB3* altered microtubule dynamics, endosomal organization, and cytokine signaling. These changes were accompanied by defects in cell adhesion, migration, and invasion. Together, our findings identify *TUBB2A* and *TUBB3* as previously unrecognized regulators of metastatic competency in breast cancer bone metastasis and support their potential utility as predictive biomarkers and therapeutic targets in this clinically challenging disease [16].

## 2. Materials and Methods

### 2.1. Antibodies

Tubulin isotypes were probed using antibodies against total α-tubulin (monoclonal; Cell Signaling Technology, Danvers, MA, USA, #3873), tubulin α1a (polyclonal; Abcam, Waltham, MA, USA, ab95966), tubulin α1b (monoclonal; Abcam, Waltham, MA, USA, ab108629), tubulin α8 (monoclonal; Novus, Minneapolis, MN, USA, NBP2-37535), tubulin β1 (monoclonal; Abcam, Waltham, MA, USA, ab179511), tubulin β2 (monoclonal; Santa Cruz, Dallas, TX, USA, sc-47751; WB), tubulin β2 (monoclonal; Abcam, Waltham, MA, USA, #ab179512; IHC), tubulin β3 (monoclonal; BioLegend 801213), tubulin β4 (monoclonal; Abcam, Waltham, MA, USA, ab179509), tubulin β6 (monoclonal; Abcam, Waltham, MA, USA, ab322871), tubulin β8 (polyclonal; Novus, Minneapolis, MN, USA, NBP3-05594), and tubulin γ1 (monoclonal; Abcam, Waltham, MA, USA, ab129021). Cell-adhesion and focal-adhesion components were detected using antibodies against integrin β1 (monoclonal; Cell Signaling Technology, Danvers, MA, USA, #9699), integrin β3 (monoclonal; Cell Signaling Technology, Danvers, MA, USA, #13166), integrin β4 (monoclonal; Cell Signaling Technology, Danvers, MA, USA, #14803), integrin β5 (monoclonal; Cell Signaling Technology, Danvers, MA, USA, #3629) integrin α4 (monoclonal; Cell Signaling Technology, Danvers, MA, USA, #8440), integrin α5 (polyclonal; Cell Signaling Technology, Danvers, MA, USA, #4705), phospho-FAK (Tyr397) (monoclonal; Invitrogen #700255), paxillin (monoclonal; Cell Signaling Technology, Danvers, MA, USA, #50195), vinculin (monoclonal, Cell Signaling Technology, Danvers, MA, USA, #13901), and talin-1 (polyclonal, Proteintech, Rosemont, IL, USA, 14168-1-AP). Endosomal and membrane-trafficking markers were detected using antibodies against Rab5 (monoclonal; Cell Signaling Technology, Danvers, MA, USA, #3547) and caveolin-1 (monoclonal; Cell Signaling Technology, Danvers, MA, USA, #3267). β-actin (monoclonal; Santa Cruz, Dallas, TX, USA, sc-47778) served as the loading control. Secondary antibodies used: HRP-conjugated goat anti-rabbit IgG (Bio-Rad Hercules, CA, USA, #1706515) and HRP-conjugated goat anti-mouse IgG (Bio-Rad Hercules, CA, USA, #1706516) at 1:3000, goat anti-mouse IgG conjugated to Alexa Fluor 594 (Invitrogen #A-11005) and goat anti-rabbit IgG conjugated to Alexa Fluor 594 (Invitrogen #A-11012) at 1:1000. Note that the tubulin β2 antibody (Santa Cruz, Dallas, TX, USA, sc-47751) recognizes both β2a (TUBB2A) and β2b (TUBB2B) isotypes. Accordingly, protein-level data derived from this antibody are labeled as “Tubulin β2” throughout figures and results.

### 2.2. Cell Culture and Cell Lines

Bone-derived MDA-MB-231 cells were isolated and cultured as previously described [2]. These cells were subsequently used for CRISPR-mediated genome editing, as described below. Cells were maintained in minimum essential medium (MEM; Corning, St. Louis, MO, USA, #10-010-CV) supplemented with 10% fetal bovine serum (FBS; Sigma-Aldrich, St. Louis, MO, USA, #F0926), 1% penicillin-streptomycin (100 U/mL penicillin, 100 µg/mL streptomycin; Gibco, Waltham, MA, USA, #15140-122), and 5 µg/mL Plasmocin prophylactic (InvivoGen #ant-mpp, San Diego, CA, USA). Cells were maintained at 37 °C in a humidified atmosphere of 5% CO_2_.

### 2.3. CRISPR-Cas9 Gene Editing

Guide RNA (gRNA) sequences targeting *TUBB2A* and *TUBB3* were designed using the GeneArt CRISPR gRNA design tool (Invitrogen) and obtained as synthetic sgRNA. The sequences were 5′-AGGTCATCAGCGATGAGCAT-3′ (TUBB2A) and 5′-GTCGCCCACGTAGTTGCCGC-3′ (TUBB3; antisense-targeting). Upon receipt, gRNAs were resuspended and quantified by NanoDrop (Thermo Fisher Scientific, Waltham, MA, USA). Editing was performed by transfection following the TrueCut Cas9 Protein v2 protocol (Invitrogen, Carlsbad, CA, USA, #MAN0017066, pp. 7–8), using the Lipofectamine CRISPRMAX Cas9 Transfection Reagent (Invitrogen, Carlsbad, CA, USA, #CMAX00003) at the 24-well scale. Per reaction, the first mixture (Tube 1) contained 25 µL Opti-MEM (Invitrogen, Carlsbad, CA, USA, #31985-070), 1.25 µg TrueCut Cas9 Protein v2 (Invitrogen, Carlsbad, CA, USA, #A36498), 240 ng gRNA, and 2.5 µL Cas9 Plus Reagent; the second mixture (Tube 2) contained 25 µL Opti-MEM and 1.5 µL Lipofectamine CRISPRMAX Reagent. The two were combined and applied to cells. Bone-derived MDA-MB-231 cells were seeded to 30–70% confluency and transfected in Opti-MEM (0% FBS), with the serum restored 4 h after transfection. At 48 h post-transfection, cells were collected, counted, and seeded for single-cell cloning at 0.8 cells/well in 96-well plates. Clones were propagated for 2–3 weeks, then expanded and screened by Western blot to assess editing of *TUBB2A* and *TUBB3*. Five clones of each were then purified for gDNA utilizing the DNeasy Blood & Tissue Kit (QIAGEN, Germantown, MD, USA, #69504) and validated by next-generation sequencing (NGS) at the Genome Research Core (GRC), University of Illinois at Chicago. Editing outcomes were quantified using CRISPResso (v2.3.2), with figures provided in the Supplementary Materials. In parallel with the TUBB2A and TUBB3 targeting guides, a TrueGuide sgRNA Negative Control (Invitrogen, Carlsbad, CA, USA, #A35526) was used to generate scramble-control lines.

### 2.4. Immunofluorescence

Immunofluorescence was performed as previously described [3]. Cells were seeded on 8-well chamber glass slides (CellTreat, Ayer, MA, USA, #229168) at a density ranging from 5000 to 10,000 cells per well. Primary antibodies were used at the following dilutions: total α-tubulin, 1:1000; Rab5, 1:200; and caveolin-1 (Cav1), 1:500. Alexa Fluor 594-conjugated anti-mouse or anti-rabbit secondary antibodies were used as appropriate (1:1000). Slides were mounted using ProLong Gold Antifade with DAPI (Cell Signaling Technology #8961). Images were acquired using a Zeiss LSM 710 confocal microscope with 10×, 20×, or 40× objectives. Microtubule regrowth images were analyzed using ImageJ (v1.53a). CellProfiler (v4.2.8) was used to analyze endosomal puncta and cellular. Fluorescent intensity quantifications were normalized to cell size (AU/µm^2^). Endosomal puncta were identified within each segmented cell using the IdentifyPrimaryObjects module and classified by diameter into small (0.5–1.0 µm), medium (1.0–2.0 µm), and large (2.0–4.0 µm) bins. The number of puncta counts per cell within each category was recorded. Data are presented in mean, error bars as SEM.

### 2.5. Microtubule Regrowth Assay

Cells were seeded in 8-well chamber slides and treated with 20 μM nocodazole (Cell Signaling Technology, Danvers, MA, USA, #2190) for 3 h on ice to induce microtubule depolymerization. Following treatment, nocodazole was removed by three high-volume washes with ice-cold PBS, ensuring complete drug clearance while preventing premature microtubule reassembly. Because tubulin polymerization is thermodynamically unfavorable at 4 °C, maintaining cells on ice effectively decoupled the drug-removal phase from the regrowth phase. Microtubule regrowth was then initiated by replacing the PBS with pre-warmed growth medium and transferring cells to 37 °C, such that polymerization was triggered exclusively by temperature recovery. Cells were fixed at 0, 5, 10, 15, 20, and 30 min after nocodazole washout using 4% paraformaldehyde (Thermo Fisher Scientific, Waltham, MA, USA, #J61899) and processed for immunofluorescence. Microtubules were labeled with α-tubulin primary antibody (1:2000) and an Alexa Fluor 594-conjugated anti-mouse secondary antibody (1:1000). Cells were imaged using a Zeiss LSM 710 confocal microscope equipped with a 40× oil-immersion objective. Microtubule aster areas (µm^2^) were quantified using the segmented line tool in ImageJ. Data are presented as fold change relative to scramble, with per-image means used for statistical analysis (*n* = 44 images, 513 total cells; two-way ANOVA with Dunnett’s multiple-comparison test).

### 2.6. Immunohistochemistry

Matched primary and bone-metastatic tumor biopsies were obtained from Rush Pathology (*n* = 16 patients). Sections were cut, deparaffinized, and rehydrated through xylene and graded ethanol baths. Because bone-metastatic sections were decalcified prior to processing, enzymatic antigen retrieval was used in place of heat-induced epitope retrieval to preserve tissue morphology and section adhesion. Antigen retrieval was performed in trypsin (Sigma-Aldrich, St. Louis, MO, USA, #T7168) at 37 °C for 30–45 min. Slides were washed in Tris-buffered saline, and endogenous peroxidase was quenched with 3% H_2_O_2_ (Thermo Fisher Scientific, Waltham, MA, USA, #H324) for 10 min at room temperature. Sections were blocked for 1 h and incubated overnight at 4 °C with primary antibody against tubulin β2 (1:5000) or tubulin β3 (1:500). Slides were then incubated with a biotinylated secondary antibody (1:300) for 30 min at room temperature. Signal was amplified with a tyramide signal amplification (TSA) kit (Akoya Biosciences, Marlborough, MA, USA, #NEL700A001KT) and developed with a DAB Peroxidase Substrate Kit (Vector Laboratories, Newark, CA, USA, #SK-4100). Stained slides were quantified in QuPath (v0.7.0). Brightfield 20× and 40× images were imported, the image type was set to brightfield (H-DAB), and color deconvolution was applied to separate the DAB and hematoxylin channels. Cells were detected with QuPath’s watershed cell-detection algorithm and classified into four categories (negative, 1+/weak, 2+/moderate, 3+/strong) using a single-measurement intensity classifier (Cell: DAB OD mean). Fixed, equal-interval thresholds (weak 0.20, moderate 0.47, strong 0.73) were applied identically to every specimen. For each ROI, an H-score was calculated as (1 × %weak) + (2 × %moderate) + (3 × %strong) (range 0–300), and ROI-level scores were averaged to yield one mean H-score per patient. Matched primary and bone-metastasis H-scores were compared across the 16 patients, and pooled cell-composition percentages (aggregated across all cells and patients within each compartment) were used to calculate the fold-change in intensity distribution between compartments.

### 2.7. Western Blotting

Whole-cell lysates were prepared in RIPA lysis and extraction buffer (Thermo Fisher Scientific, Waltham, MA, USA, #89900) supplemented with EDTA-free protease inhibitor cocktail (one tablet per 10 mL; Roche #04693132001). Protein concentrations were determined by BCA assay (Thermo Fisher Scientific, Waltham, MA, USA, #23225). Lysates were normalized and combined with 4× Laemmli sample buffer (Bio-Rad, Hercules, CA, USA, #1610747) supplemented with 2-mercaptoethanol (9:1). Samples were then denatured at 100 °C for 7 min and between 2.5 and 5.0 µg of protein loaded per lane and resolved on 8–12% polyacrylamide gels (according to protein molecular weight) in SDS running buffer at 125 V for 60–90 min. Proteins were wet-transferred onto a 0.45 µm PVDF membrane (Millipore Sigma, Burlington, MA, USA, #IPVH00010) at 200 mA for 90–120 min. Membranes were blocked in 2% BSA/TBST for 30 min room temperature and then incubated with primary antibodies diluted in 2% BSA/TBST overnight at 4 °C. Dilution factors for all primary antibodies were within a range of 1:2000–1:5000. The following day, membranes were washed and incubated with the appropriate HRP-conjugated secondary antibody in 2% BSA/TBST for 60 min at room temperature. Bands were visualized using SuperSignal West Pico PLUS (Thermo Scientific, Waltham, MA, USA, #34580) or SuperSignal West Dura (Thermo Scientific, Waltham, MA, USA, #34075) chemiluminescent substrate and imaged on an Invitrogen iBright CL1500 Imaging System (Thermo Fisher Scientific, Waltham, MA, USA). Intensities were quantified using GelAnalyzer (v23.1.1) with β-actin as the loading control. Data is presented as mean fold change relative to the scramble control from *n* = 3–5 biological samples—one-way ANOVA with Dunnett’s multiple-comparison test.

### 2.8. Trans Well Assay for Cell Migration

Cell migration was assessed using the QCM Chemotaxis 24-Well Cell Migration Assay (8 µm pore size; Millipore Sigma, Burlington, MA, USA, #ECM508). Cells (2 × 10^5^ in 200 µL serum-free MEM) were seeded into the upper chamber of the transwell inserts, and 500 µL of MEM containing 10% FBS was added to the lower chamber as a chemoattractant. Cells were allowed to migrate for 6 h (37 °C, 5% CO_2_) then stained with crystal violet. Non-migrated cells were removed from the upper surface of the membrane with a cotton swab. Cells that had migrated were imaged under a light microscope, and the retained stain extracted and absorbance read at 540 nm. Migration data is expressed as fold change relative to scramble control (*n* = 4 independent experiments; one-way ANOVA with Dunnett’s multiple-comparison test).

### 2.9. Cell Invasion Assay

Transwell inserts (24-well, 8.0 µm pore; Corning, St. Louis, MO, USA, #3422) were coated with Growth Factor Reduced Matrigel (Corning, St. Louis, MO, USA, #356231) diluted 1:3 in serum-free medium and allowed to gel at 37 °C for 30 min. Cells were serum-starved overnight in medium containing 0.25% FBS, trypsinized, and 100,000 cells seeded per insert in serum-free medium. The lower chambers were filled with medium containing 10% FBS as a chemoattractant, and the plates were incubated for 24 h (37 °C, 5% CO_2_). Non-invaded cells were removed from the upper membrane surface with a cotton swab, and invaded cells stained with crystal violet. For quantification, crystal violet was eluted in 10% acetic acid and absorbance measured. Invasion data is expressed as fold change relative to scramble (no test applied, *n* = 2 independent experiments).

### 2.10. Cell Cycle Analysis

Cells were cultured to 40–60% confluency, then trypsinized, pelleted, rinsed in PBS, and fixed with 70% ethanol at 4 °C overnight. Fixed cells were then resuspended in 0.6 mL of PI/RNase staining buffer (BD Biosciences, San Jose, CA, USA, #550825) and incubated for 15 min at room temperature in the dark. Cells were characterized on BD FACS Fortessa. Data was analyzed in FlowJo (v10.10.0), and the proportions of cells in sub-G1, G1, S, and G2/M were determined. Cell cycle data are expressed as fold change relative to scramble (*n* = 4 independent experiments; two-way ANOVA with Dunnett’s multiple-comparison test).

### 2.11. ELISA for IL6 Secretion

Cells were grown to 70% confluency under standard culture conditions. The medium was then removed, cells were rinsed with PBS and replaced with MEM containing 0.25% FBS for 16 h (37 °C, 5% CO_2_). Conditioned media was collected, centrifuged to remove debris, and secreted IL-6 levels measured using the Human IL-6 DuoSet ELISA kit (R&D System, Minneapolis, MN, USA, #DY008), according to the manufacturer’s protocol. Absorbance was read at 450 nm, and IL-6 concentrations interpolated from a recombinant human IL-6 standard curve. Data are expressed as fold change relative to scramble (*n* = 6 independent experiments; one-way ANOVA with Dunnett’s multiple-comparison test).

### 2.12. Cell Adhesion Assay

Adhesion was compared across three surfaces in a 12-well format: uncoated tissue-culture-treated plastic (CELLTREAT, Ayer, MA, USA, #229111), pre-coated collagen I (Corning BioCoat, St. Louis, MO, USA, #356500), and fibronectin-coated plastic. For the fibronectin condition, human fibronectin (Corning, St. Louis, MO, USA, #356008) was diluted to a working concentration of 10 µg/mL, applied to plastic plates, sealed with Parafilm, and incubated for 1 day at 4 °C. Coated plates were then aspirated and air-dried for ~30 min immediately before use. Cells were counted, diluted to 1 × 10^6^ cells/mL, and seeded as a 50 µL droplet (~50,000 cells) per well. Plates were incubated at 37 °C in a 5% CO_2_ incubator for 2 h, then rinsed with PBS to remove non-adherent cells. Adherent cells were stained with crystal violet (Sigma-Aldrich, St. Louis, MO, USA, #94448) for 30 min, rinsed gently under running tap water for 1–2 min, and air-dried. Plates were scanned on a flatbed scanner, and cells were imaged on a light microscope. The retained stain was then solubilized in 10% acetic acid (Millipore Sigma, Burlington, MA, USA, #AX0073) for 15 min at room temperature with shaking, and absorbance was measured at 560 nm to quantify adhesion. Data are presented as fold change relative to the scramble control (*n* = 3–4 independent experiments; one-way ANOVA with Dunnett’s multiple-comparison test).

### 2.13. Survival Kaplan–Meier and Gene DE Analysis

Kaplan–Meier Relapse-Free Survival analysis in breast cancer patients was generated using Kaplan–Meier plotted https://kmplot.com/analysis/ (accessed on 20 January 2026) [17]. The dataset included 4929 samples from 50 datasets with overall survival data. The average follow-up was 60 months. We also analyzed the Timer 2.0 dataset (Gene DE module, accessed on 14 January 2026) to study the differential expression between breast-invasive carcinoma (BRCA) and adjacent normal tissues for tubulin α and β subunits genes [18]. Distributions of gene expression levels are displayed using box plots. The statistical significance was computed by differential analysis (edgeR, version 3.2.4) on RNA-Seq raw counts and is annotated by the number of asterisks (* *p* < 0.05; ** *p* < 0.01; *** *p* < 0.001). 

### 2.14. qPCR

Total RNA was isolated from cells using Total RNA Mini Kit (IBI Scientific #IB47322) and quantified using a NanoDrop spectrophotometer, (Thermo Fisher Scientific, Waltham, MA, USA). A total of 1 µg of total RNA was reverse-transcribed into cDNA using the SuperScript III First-Strand Synthesis System for RT-PCR (Invitrogen, Carlsbad, CA, USA, #18080051), with random hexamers, and according to the manufacturer’s instructions. The resulting cDNA was diluted 1:10 in molecular-grade water and used for quantitative PCR with SYBR-based master mix (Accuris, Edison, NJ, USA, #PR2000) on a QuantStudio 5 Real-Time PCR System (Applied Biosystems, Waltham, MA, USA). Reactions were performed in technical duplicate; duplicates with a Ct difference > 0.5 were excluded. Cycling conditions: polymerase activation at 95 °C for 10 min, followed by 40 cycles of 95 °C for 15 s and 60 °C for 60 s, with a final melt-curve analysis from 60 °C to 95 °C. Single peaks were observed for all primer pairs. Relative gene expression was calculated using the comparative Ct (2^−ΔΔCt^) method and normalized to the mean of two reference genes (*GAPDH* and *ACTB*). Data is represented as fold change relative to scramble (*n* = 4 independent experiments; two-way ANOVA with Dunnett’s multiple-comparison test).

### 2.15. RNA Sequencing and Analysis

To define transcriptional programs associated with β-tubulin isotype depletion, we performed exploratory RNA sequencing (RNA-seq)-based transcriptomic profiling of TUBB2A knockout (KO) and TUBB3 knockdown (KD) MDA-MB-231 cells. Because the RNA-seq experiment was conducted using a single library per experimental group (*n* = 1), the analysis was intended as an exploratory, hypothesis-generating approach to identify candidate genes and pathways associated with TUBB2A and TUBB3 depletion for subsequent validation. Total RNA was isolated from cells in-house using Total RNA Mini Kit (IBI Scientific, Dubuque, IA, USA, #IB47322). Library preparation, sequencing, and bioinformatic analysis were performed by Novogene (Sacramento, CA, USA). Poly(A) + RNA was enriched using oligo(dT) magnetic beads, fragmented, and converted into strand-specific (dUTP) cDNA libraries by end repair, A-tailing, adapter ligation, size selection, and PCR amplification. Libraries were sequenced on an Illumina NovaSeq X Plus (paired-end, 150 bp, Illumina, San Diego, CA, USA), generating 58.4–63.1 million clean reads (29.2–31.6 million read pairs) per sample (Q30 ≥ 96.2%, error rate 0.01%, GC content 48.6–48.7%). Raw reads were filtered with fastp and clean reads aligned to the human reference genome (GRCh38) with HISAT2 (v2.2.1), with 97.7–97.8% total and 93.2–93.5% unique mapping rates. Gene-level counts were generated with featureCounts (v2.1.1), and expression levels reported as fragments per kilobase of transcript per million mapped reads (FPKM). Differential expression analysis was performed on raw counts using edgeR (v4.0.16) with a single library per group (*n* = 1). Library sizes were normalized by TMM (calcNormFactors). Because biological dispersion cannot be estimated without replication, a fixed common dispersion of 0.04 (BCV = 0.2) was applied to all genes, as recommended in the edgeR User’s Guide. Each knockout/knockdown line was compared with the scramble control separately using the two-sided exact test. Genes with a Benjamini–Hochberg-adjusted *p* ≤ 0.05 and |log2FC| ≥ 1.0 were used to rank and prioritize candidates for exploratory analysis. GO, KEGG, and Reactome enrichment analyses of up- and downregulated candidate genes were performed with clusterProfiler (v4.8.1) with Benjamini–Hochberg-adjusted *p* ≤ 0.05 considered significant.

### 2.16. Statistical Analysis

Graphs and statistical analyses were generated in GraphPad Prism (v10.4.1). Comparisons among scramble, TUBB2A KO, and TUBB3 KD cells used one-way or two-way ANOVA as appropriate, followed by Dunnett’s multiple-comparisons test against the scramble control. For imaging-based quantification, individual cells were segmented and quantified via CellProfiler or ImageJ, while the per-image mean was used as the unit of replication. Significance was defined as *p* < 0.05. Statistical levels were categorized as follows: (* *p* < 0.05; ** *p* < 0.01; *** *p* < 0.001; **** *p* < 0.0001). Exact tests and significance markers are indicated in the corresponding figure legends. Statistical analysis of RNAseq data is described separately.

## 3. Results

### 3.1. Elevated TUBB2A and TUBB3 Expression Correlates with Poor Clinical Outcomes in Breast Cancer

**Figure 1:**
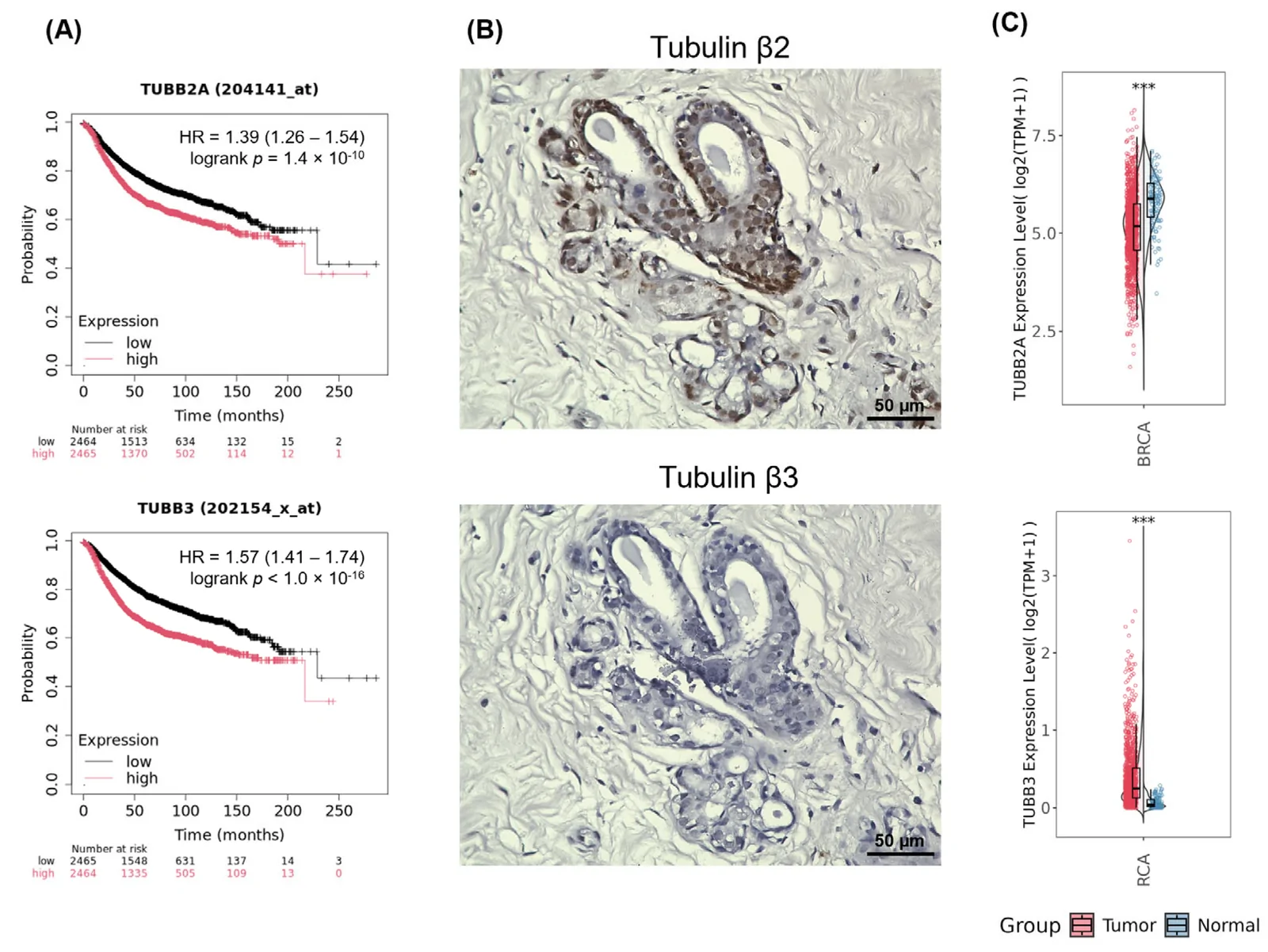
Clinical significance of the *TUBB2A* and *TUBB3* target genes in breast cancer patients. (**A**) Kaplan–Meier plots correlating gene-chip mRNA expression of *TUBB2A* and *TUBB3* to the survival of breast cancer patients (*n* = 4929). High expression of both isotypes was associated with worse survival (*TUBB2A*, HR 1.39; *TUBB3*, HR 1.57). Plots were generated using KM Plotter (https://kmplot.com/analysis/, accessed on 20 January 2026), including all breast cancer subtypes without restriction by treatment. Patients stratified at the median. (**B**) Immunohistochemistry of normal human mammary gland tissue sections, stained for tubulin β2 and β3. Strong β2 staining but not β3 was observed in the epithelial lining cells of the mammary ducts. Representative of 5 images per marker. Images captured at 40×, scale bar 50 µm. (**C**) Expression of *TUBB2A* and *TUBB3* (log_2_[TPM + 1]) in BRCA tumors (red) versus normal (blue) breast tissue. Tumor samples demonstrated significant downregulation of *TUBB2A* and upregulation of *TUBB3* (*n* = 1093 tumor, *n* = 112 normal; *** *p* < 0.001). Analyzed using TIMER2.0 (http://timer.cistrome.org/, accessed on 14 January 2026). TPM: Transcripts per million.

To evaluate the clinical relevance of tubulin isotype expression in breast cancer, we analyzed relapse-free survival using the Kaplan–Meier Plotter database based on mRNA expression of α-, β-, γ-, δ-, and ε- tubulin isotypes (Supplementary Figure S1). All available breast cancer cases with relapse-free survival data (*n* = 4929) were included, irrespective of receptor status or treatment history. Higher expression of *TUBB2A* (HR = 1.39), *TUBB3* (HR = 1.57), *TUBΒ4B* (annotated *TUBΒ2C* in KM Plotter) (HR = 1.51), and *TUBB6* (HR = 1.30) were significantly associated with reduced relapse-free survival (logrank *p* < 0.0001), indicating poorer clinical outcomes. In contrast, higher *TUBΒ1* expression correlated with improved prognosis (HR = 0.66), while *TUBΒ2B* showed only minimal association with survival (HR = 0.90) (Figure 1A and Supplementary Figure S1A). Associations with relapse-free survival were not restricted to the β-tubulins. Among α-tubulin isotypes, higher expression of *TUBA1A* (HR = 1.24), *TUBA1B* (HR = 1.26), *TUBA1C* (HR = 1.37), and *TUBA4A* (HR = 1.23) was associated with reduced relapse-free survival, whereas higher expression of *TUBA3C* (HR = 0.83), *TUBA3D* (HR = 0.87), *TUBA4B* (HR = 0.79), and *TUBA8* (HR = 0.77) was associated with improved survival (Supplementary Figure S1B,C). This suggests that specific tubulin isotypes contribute to disease progression.

To further examine expression patterns, we assessed tubulin β2 and tubulin β3 protein abundance using IHC in normal mammary tissue, and mRNA expression using a publicly available TIMER 2.0 dataset for breast-invasive carcinoma. In normal mammary tissue, tubulin β2a staining was greater than tubulin β3 as examined via IHC (Figure 1B). However, in primary breast tumors, *TUBB2A* expression was reduced, whereas *TUBB3* expression was significantly increased as examined via the TIMER 2.0 dataset (Figure 1C).

Consistent with these observations, several α-tubulin isotypes (*TUBA1B*, *TUBA1C*, *TUBA3D*, and *TUBA4D*) were upregulated in tumors compared to normal tissue, while *TUBA1A* expression was decreased (Supplementary Figure S2). Collectively, these findings identify *TUBB2A* and *TUBB3* as clinically relevant and dynamically regulated β-tubulin isotypes in breast cancer, each showing a strong association with poor patient outcomes despite their distinct expression patterns between normal and tumor tissues.

### 3.2. Increased Abundance of Tubulin β2 and Tubulin β3 in Breast Cancer Bone-Metastatic Tumors

**Figure 2:**
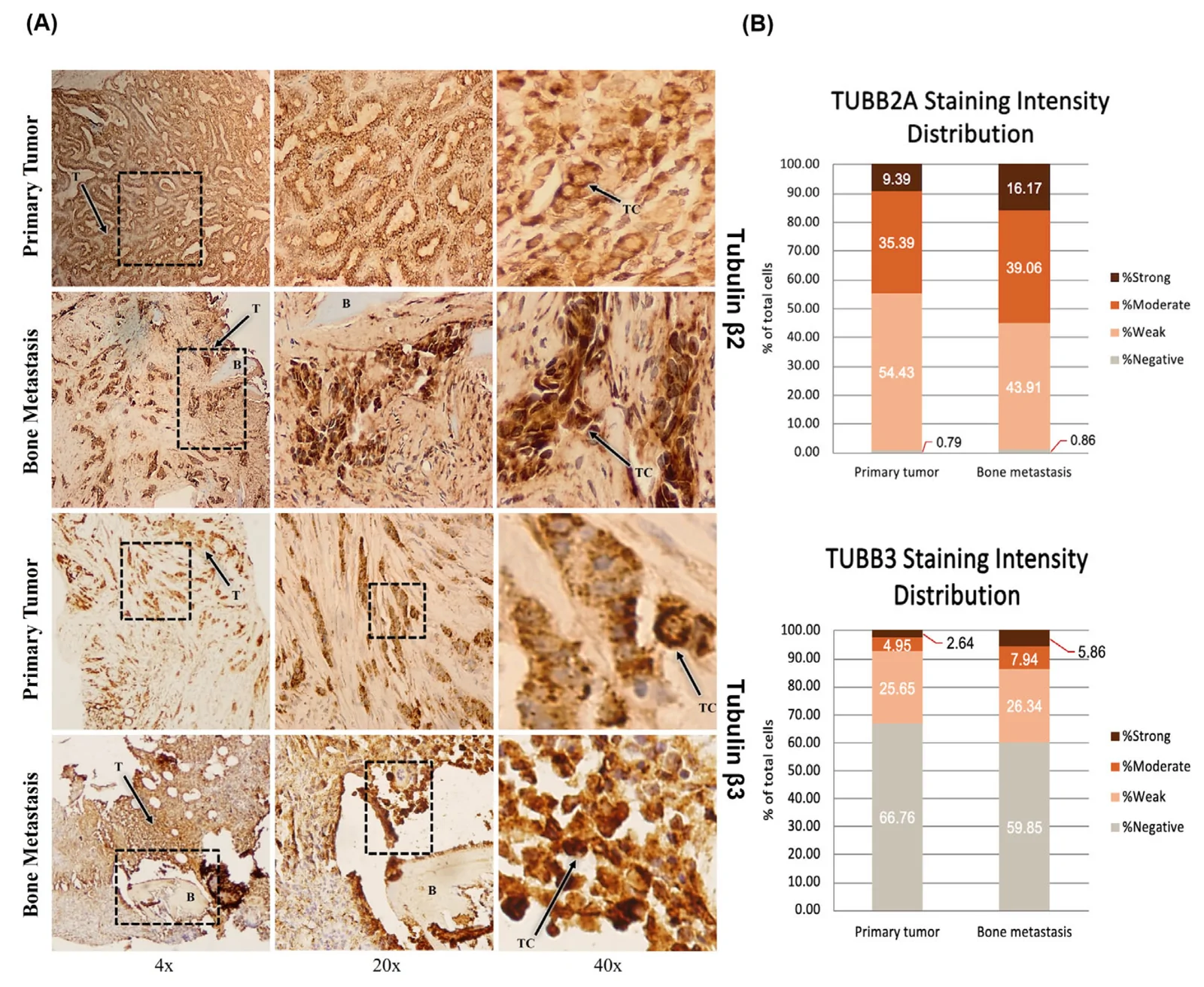
Tubulin β2 and β3 abundance in matched bone-metastatic tumors. (**A**) Immunohistochemistry assessing differential staining of microtubule isotypes between matched primary and bone-metastatic breast tumors (*n* = 16). From left to right are 4×, 20×, and 40× images. Dashed squares represent areas captured at higher magnifications. Symbols shown are identified as: T (tumor), B (bone), TC (tumor cell). Stained for tubulin β2 top (Abcam #ab179512) and tubulin β3 bottom (Biolegend #801213). (**B**) Analyzed 269 images. Tubulin β2 (237 40× images) and tubulin β3 (32 20× images) staining intensity distribution in primary breast tumors versus matched bone-metastatic lesions (*n* = 16 patients). Stacked bars depict the percentage of pooled cells (all patients combined) separated into each of the four staining-intensity categories (Negative, Weak, Moderate, Strong). Statistical analysis performed using two-way ANOVA demonstrated a main effect of tissue type (primary vs. bone metastasis; *p* = 0.03) for tubulin β2, but no significant main effect for tubulin β3. IHC analysis revealed increased tubulin β2 and tubulin-β3-positive cell populations in bone-metastatic lesions relative to primary breast tumors, with strongly positive cells elevated 1.7-fold (9.4% vs. 16.2%) in tubulin β2 and 2.22-fold (2.6% vs. 5.9%) in tubulin β3.

Based on breast cancer patient-survival analyses, we next evaluated tubulin β2 and tubulin β3 expression in bone-metastatic breast cancer tissues. IHC analysis of matched patient samples (*n* = 16) revealed that tubulin-β2-strongly-positive cells were elevated approximately 1.7-fold in bone-metastatic lesions compared with primary breast cancer tumors (9.4% vs. 16.2% of total cells; Figure 2A,B). Quantitative assessment of tubulin-β3-positive cells revealed an increase in moderate (1.60-fold; 5.0% vs. 7.9%) and strongly positive (2.22-fold; 2.6% vs. 5.9%) cells in bone-metastatic tumors compared to primary tumors, with no substantial change in the overall weakly positive fraction (25.6% vs. 26.3%) or total positive-cell burden between bone metastasis and primary tumor samples (Figure 2B). These findings indicate that cells within bone-metastatic lesions carry higher β2- and β3-tubulin protein burden, suggesting a metastasis-stage-specific role for these β-tubulin isotypes in promoting tumor cell adaptation to the bone microenvironment. The enrichment of high-intensity tubulin β2 and tubulin β3 staining in bone-metastatic lesions further highlights their utility as markers of bone-metastatic progression in breast cancer.

### 3.3. TUBB2A Knockout and TUBB3 Knockdown Alters Mitotic Progression and Delays Microtubule Regrowth

**Figure 3:**
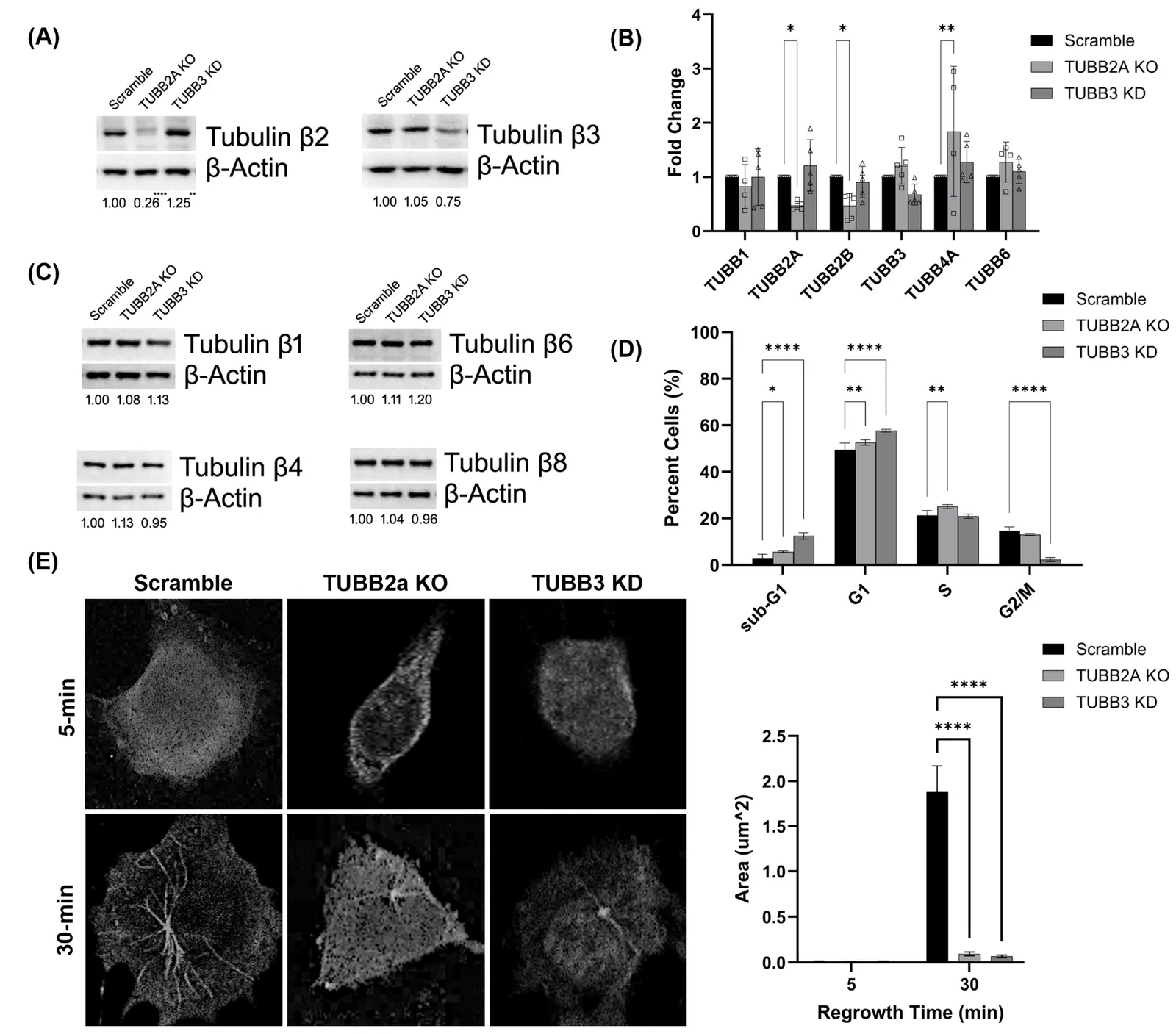
Generation and characterization of tubulin β2a- and β3-deficient cells. (**A**) Western blot analysis of tubulin β2a and tubulin β3 expression in scramble, TUBB2A KO, and TUBB3 KD cells. Tubulin β2a was markedly reduced in TUBB2A KO cells (**** *p* < 0.0001, *n* = 4), while tubulin β3 showed a modest reduction in TUBB3 KD cells (*p* = 0.1171, *n* = 3). Quantification represents fold change relative to scramble with β-actin serving as a loading control. One-way ANOVA. (**B**) Quantitative PCR analysis comparing β-tubulin isotype expression (*TUBΒ1*, *TUBB2A*, *TUBΒ2B*, *TUBB3*, *TUBB4A*, and *TUBΒ6*). Gene expression was calculated using the ΔΔCt method normalized to an average of 2 housekeeping genes (ACTB and GAPDH) and is presented as fold change relative to scramble. TUBB2A KO cells demonstrated reduced *TUBB2A* and *TUBΒ2B* with increased *TUBB4A*. Bars represent mean ± standard deviation; symbols represent individual biological samples (*n* = 4–5). Two-way ANOVA. (**C**) Western blot analysis of β-tubulin isotype protein expression (tubulin β1, β4, β6, β8) revealed no significant changes. Quantification represents fold change relative to scramble (*n* = 4) with β-actin serving as loading control. One-way ANOVA. (**D**) Cell cycle distribution was assessed by flow cytometry quantified across sub-G1, G1, S, and G2/M populations. TUBB3 KD cells showed a significant reduction in the G2/M population relative to scramble control, with an accumulation in the sub-G1 and G1 populations, consistent with impaired progression through cell division. Data represent mean ± SD of *n* = 4 independent experiments. Two-way ANOVA. (**E**) Microtubule regrowth assay demonstrated impaired regrowth in both TUBB2A KO and TUBB3 KD cells. Representative images at 5- and 30 min recovery (**left**) and ImageJ quantification of mean aster area per image (**right**). At 5 min, aster area was comparable across all genotypes indicating intact nucleation. At 30 min, regrowth was severely impaired in TUBB2A KO (~5% of scramble) and TUBB3 KD (~4% of scramble). Aster area was measured in a total of 513 cells across 44 images (Scramble, 174; TUBB2A KO, 147; TUBB3 KD, 192). Per-image means were used as the unit of analysis. Bar graph represents mean ± SD of per-image means. Two-way ANOVA. All ANOVA analyses were performed with Dunnett’s multiple-comparison analysis. All statistical levels are denoted as follows: * *p* < 0.05, ** *p* < 0.01, **** *p* < 0.0001. For original WB Figures please see Supplementary File S1.

To investigate the functional roles of *TUBB2A* and *TUBB3*, we generated loss-of-function models in bone-derived MDA-MB-231 cells using CRISPR/Cas9-mediated gene editing. Successful knockout of *TUBB2A* (TUBB2A KO; 96% of reads carrying a frameshifting indel) and knockdown of *TUBB3* (TUBB3 KD; ~33% of reads with an indel) was confirmed by next-generation sequencing (NGS), as described in the Methods (Supplementary Figure S3). Immunoblot analysis revealed a ~75% reduction in tubulin β2 protein levels and approximately 25% reduction in tubulin β3 protein levels compared with control cells (Figure 3A). To assess potential compensatory changes, qPCR analysis of β-tubulin isotypes was performed. These results showed a significant increase in *TUBB4A* expression in TUBB2A KO cells (1.8-fold vs. Scramble; ** *p* < 0.01), whereas the expression of other isotypes was not significantly altered (Figure 3B and Supplementary Figure S5A,B). Immunoblot analysis showed no significant changes in the protein levels of β-tubulin isotypes in TUBB2A KO and TUBB3 KD cells (Figure 3C). γ-Tubulin, by contrast, was modestly elevated in TUBB3 KD cells (1.36-fold vs. Scramble; * *p* < 0.05), with no corresponding change in TUBB2A KO cells.

Flow cytometric analysis of DNA content revealed a marked redistribution of the cell cycle in TUBB3 KD cells (Figure 3D and Supplementary Figure S4A). The G2/M fraction was reduced from 14.7% in Scramble to 2.3% (*p* < 0.01), with a corresponding increase in G1 (49.4% to 57.7%), whereas the S-phase fraction was unchanged (21.3% vs. 20.9%). Sub-G1 events were elevated from 3.0% to 12.5%. The selective depletion of G2/M, accompanied by an increased sub-G1 population, suggests that TUBB3 KD cells are lost at or shortly after mitotic entry.

Consistent with these findings, immunofluorescence analysis revealed more prominent spindle-associated β3-tubulin staining than β2-tubulin during mitosis (Supplementary Figure S4B). While differential staining intensity does not by itself establish a unique functional requirement for TUBB3 in spindle assembly, it suggests that TUBB3 may be more abundantly incorporated into the mitotic spindle, potentially contributing to the selective G2/M defects observed following TUBB3 depletion.

In contrast, TUBB2A KO cells showed no reduction in G2/M (13.1% vs. 14.7% in scramble), with significant but more modest changes in sub-G1 (5.6% vs. 3.0%), G1 (52.6% vs. 49.4%), and S (25.1% vs. 21.3%). Together, these data indicate that *TUBB3* knockdown selectively depletes the G2/M population with an accompanying rise in sub-G1 events, whereas *TUBB2A* loss produces only a modest redistribution across the cell cycle.

Next, we examined whether depletion of *TUBB2A* or *TUBB3* alters MT dynamics. Microtubule regrowth assays were performed following cold treatment and nocodazole-induced depolymerization, followed by drug washout. Control cells exhibited robust MT regrowth with near-complete recovery within 30 min. In contrast, both TUBB2A KO and TUBB3 KD cells showed markedly delayed microtubule repolymerization and failed to achieve comparable recovery even at 30 min post-washout (Figure 3E and Supplementary Figure S6A,B). To assess morphological differences, we measured the aspect ratio of each cell (major axis length divided by minor axis length) using CellProfiler. TUBB3 KD cells showed a significantly greater aspect ratio than scramble (2.24 ± 0.11 vs. 1.74 ± 0.06; *** *p* < 0.001) (Supplementary Figure S4C), confirming our qualitative observation that these cells appear more elongated in culture. TUBB2A KO cells, which occasionally appeared more spread and circular, showed no significant differences in morphology measures relative to scramble.

**Figure 4:**
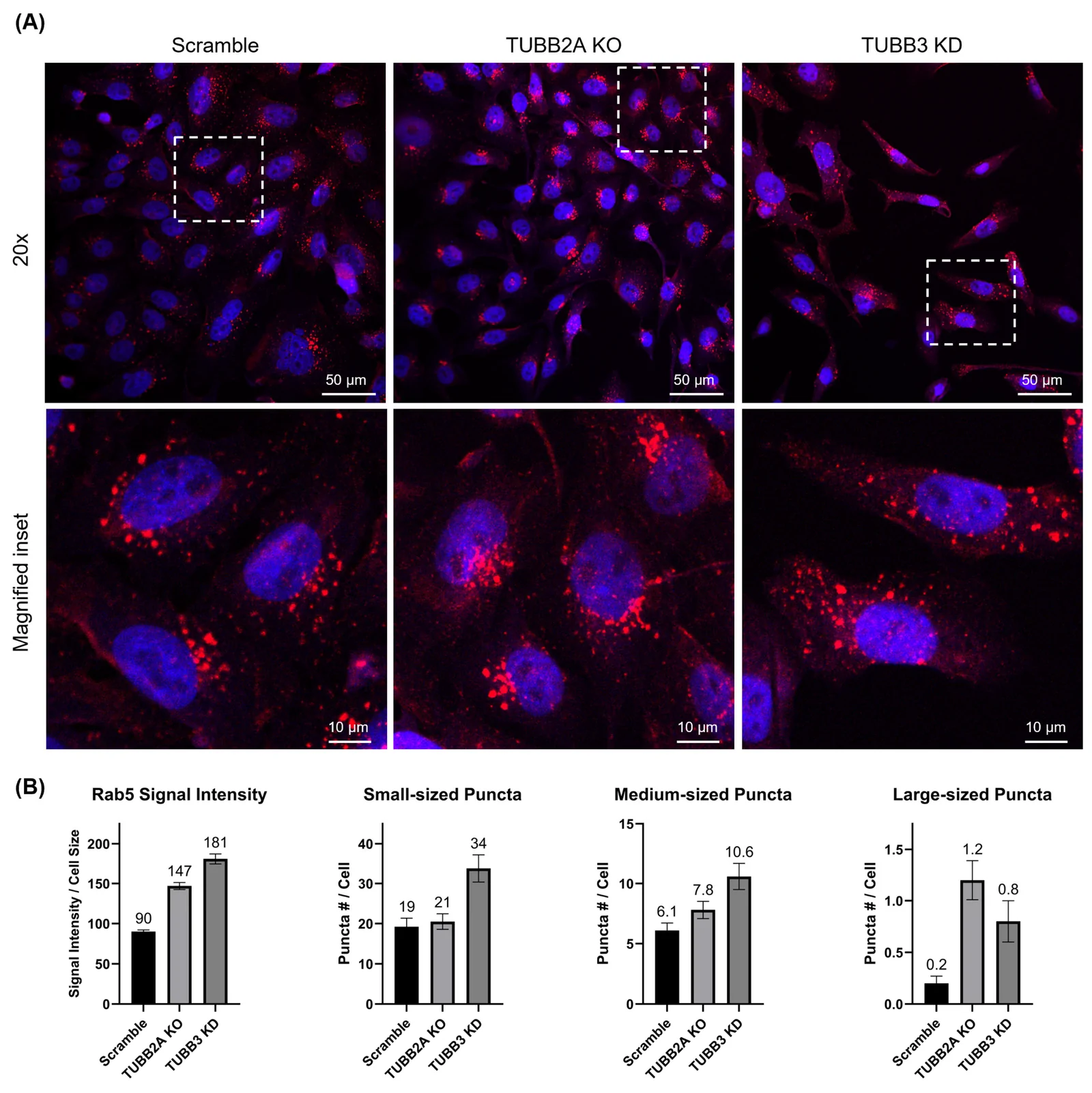

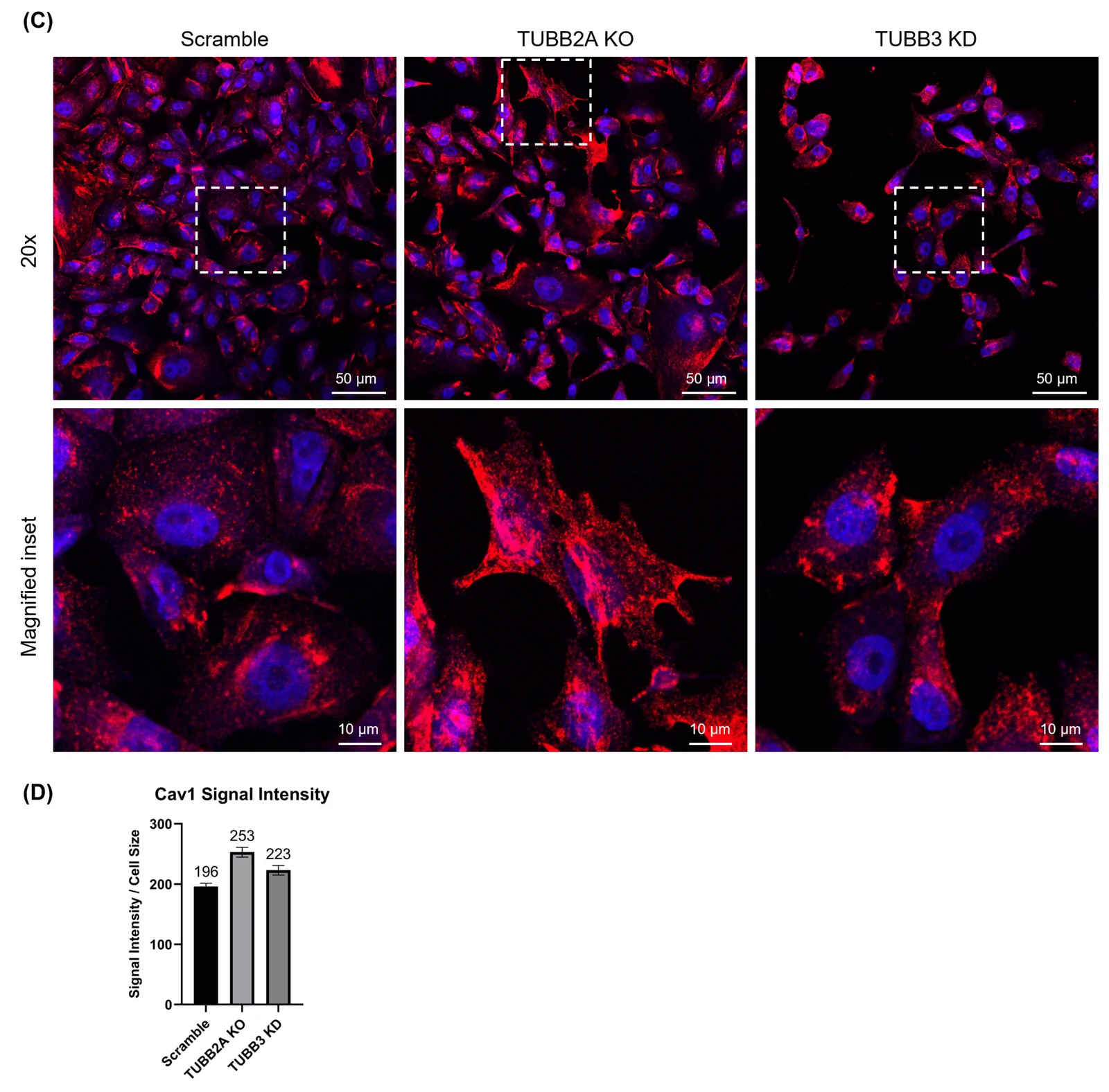
Loss of *TUBB2A* and partial depletion of *TUBB3* alter baseline Rab5- and Cav1-associated endocytic structures. (**A**) Representative confocal immunofluorescence images of Rab5-positive early endosomes (red) and DAPI-stained nuclei (blue) in scramble control, TUBB2A KO, and TUBB3 KD cells, acquired with a 20× objective. Dashed boxes indicate regions digitally magnified to highlight differences in Rab5-positive structures, with corresponding zoomed insets shown below each image. (**B**) Quantification of Rab5 signal intensity normalized to cell area, and of Rab5-positive puncta binned by diameter as small (0.5–1.0 µm), medium (1.0–2.0 µm), or large (2.0–4.0 µm). Both TUBB2A KO and TUBB3 KD cells showed increased large puncta per cell relative to scramble (6-fold and 4-fold, respectively). TUBB3 KD cells additionally showed increased Rab5 signal intensity (2-fold) and increased small- and medium-sized puncta. Data represent per-cell means from 195 total cells across all groups. Error bars indicate SEM. (**C**) Representative immunofluorescence images of Caveolin-1 (Cav1, red) and DAPI-stained nuclei (blue). (**D**) Quantification of Cav1 signal intensity normalized to cell area. TUBB2A KO cells showed a modest increase relative to scramble ~1.25 fold. Data represent means from 367 total cells, all groups. Error bars indicate SEM.

Collectively, these results demonstrate that loss of *TUBB2A* and partial depletion of *TUBB3* alters mitotic progression and disrupts microtubule regrowth, highlighting their critical roles in regulating microtubule dynamics and cell-cycle progression in bone-metastatic breast cancer cells (Figure 4).

### 3.4. TUBB2A KO and TUBB3 KD Disrupts Endosomal Subcellular Distribution

Microtubules act as tracks for endosomal trafficking, enabling the movement of endosomes between the cell periphery and the perinuclear region. To assess whether loss of *TUBB2A* or partial depletion of *TUBB3* affects endosomal organization, we examined the localization of the early endosomal marker rab5 and the caveolar marker caveolin-1 (Cav1) by immunofluorescence microscopy. Rab5 staining in scramble control cells showed distinct puncta concentrated within a characteristic perinuclear endosomal cloud (Figure 4A). In TUBB2A KO cells, we observed enhanced clustering of the perinuclear region and a pronounced puncta gradient extending toward the cell periphery in TUBB3 KD cells (Figure 4A). Quantitative analysis of knockout/knockdown cells demonstrated elevated rab5 intensity relative to scramble (1.6-fold in TUBB2A KO, 2.0-fold in TUBB3 KD), indicating an accumulation of rab5-positive endosomal structures following *TUBB2A* and *TUBB3* depletion (Figure 4B). Size-based analysis further revealed these cells exhibited greater abundance of enlarged rab5-positive puncta relative to scramble (6-fold in TUBB2A KO, 4-fold in TUBB3 KD), whereas the quantity of smaller puncta remained relatively unchanged (Figure 4B and Supplementary Figure S7). Cav1 fluorescence intensity was modestly elevated in both knockout/knockdown conditions relative to scramble controls, consistent with altered caveolar/endosomal organization and increased accumulation of Cav1-positive vesicular compartments (Figure 4C,D). Collectively, these findings suggest that depletion of either *TUBB2A* or *TUBB3* disrupts MT-dependent endosomal distribution, leading to enlargement, and spatial redistribution of rab5-positive vesicles. These results support a requirement for β-tubulin isotypes in maintaining proper subcellular endosomal organization.

### 3.5. Transcriptional Reprogramming Induced by TUBB2A Knockout or TUBB3 Knockdown

**Figure 5:**
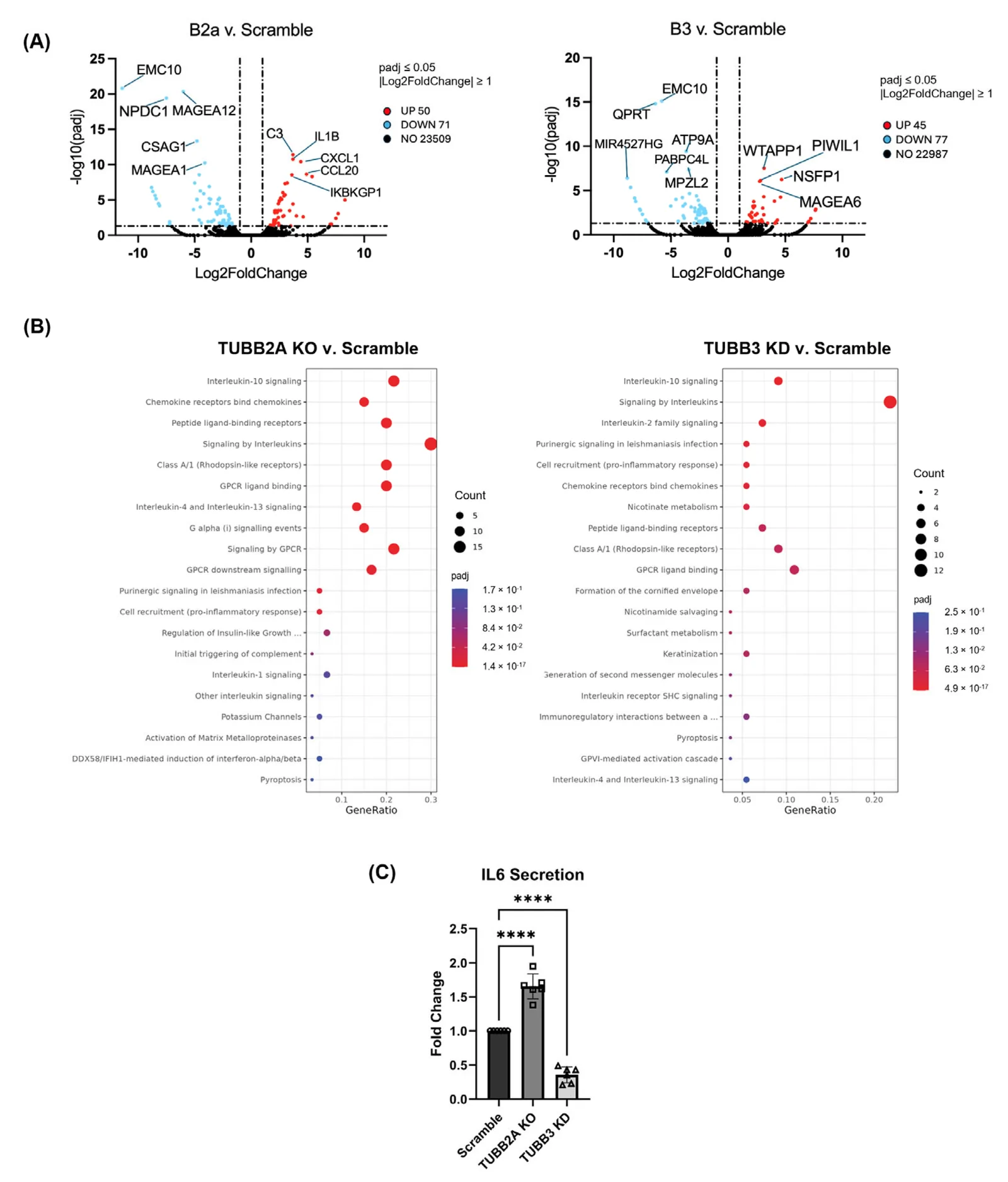
Transcriptomic profiling of TUBB2A KO and TUBB3 KD bone-metastatic MDA-MB-231 cells. (**A**) Volcano plots of differential gene expression in TUBB2A KO (left) and TUBB3 KD (right) cells relative to scramble control. Each point represents a gene. Dashed lines indicate significance thresholds of |log2FC| ≥ 1 (vertical) and adjusted p ≤ 0.05 (horizontal). Genes meeting both thresholds are shown in red (upregulated) and blue (downregulated), identifying 121 and 122 genes, respectively. Differential expression was assessed using edgeR with *n* = 1 biological replicate per genotype; because biological dispersion cannot be estimated without replication, a fixed common dispersion of 0.04 (BCV = 0.2) was applied. (**B**) Reactome pathway enrichment analysis of differentially expressed genes in TUBB2A KO (left) and TUBB3 KD (right) cells. Pathways are ranked by adjusted *p*-value. Dot size represents the number of differentially expressed genes and dot color represents adjusted *p*-value. (**C**) ELISA quantification of IL-6 secretion in conditioned media from scramble, TUBB2A KO, and TUBB3 KD cells. IL-6 secretion was increased in TUBB2A KO cells (1.7-fold, **** *p* < 0.0001) and reduced in TUBB3 KD cells (0.4-fold, **** *p* < 0.0001) relative to scramble. Bars represent mean ± SD, symbols represent individual biological samples (*n* = 6). One-way ANOVA with Dunnett’s multiple-comparisons test.

Alterations in microtubule dynamics, stability, or composition reshape intracellular signaling and nuclear trafficking of key transcriptional regulators, thereby directly influencing gene expression programs. To define transcriptional programs associated with the loss or depletion of β-tubulin isotypes, we performed discovery RNA-sequencing-based transcriptomic profiling of TUBB2A KO and TUBB3 KD MDA-MB-231 cells. Differential expression analysis identified 121 genes in TUBB2A KO cells (50 upregulated, 71 downregulated) and 122 genes in TUBB3 KD cells (45 upregulated, 77 downregulated) meeting thresholds of |log2FC| ≥ 1 and adjusted *p* ≤ 0.05 relative to scramble controls (Figure 5A). To independently assess selected candidates, we performed qRT-PCR for a panel of top RNAseq-nominated gene (IL1B, ATP9A, HAS2, EPHB3, GABRA3) in independent biological samples (*n* = 3). qRT-PCR confirmed the direction and relative magnitude of the RNA-seq identified changes for all tested candidates (Supplementary Figure S8B).

Reactome enrichment analysis and Gene Ontology analyses suggested prominent modulation of cytokine–cytokine receptor interaction pathways, with enrichment of IL-10-associated inflammatory signaling, implicating β-tubulin-dependent cytoskeletal regulation in control of inflammatory and secretory programs (Figure 5B and Supplementary Figure S8A). Notably, TUBB2A KO cells exhibited a transcriptional signature of an NF-κB/IRF-driven inflammatory module, anchored by the osteoclastogenic cytokines IL-1β and IL-6 and accompanied by an increased expression of CXCR2-ligand chemokines. This suggests a transcriptional program consistent with a pro-osteolytic inflammatory phenotype. Across both TUBB2A KO and TUBB3 KD cells, *EMC10* emerged as the most strongly altered transcript, suggesting a shared tubulin-isotype-sensitive regulatory node. Among the top 100 differentially expressed genes, *MMP1* was the most highly expressed and increased in both TUBB2A KO and TUBB3 KD cells. Additional inflammatory and secretory genes, including *CSF3*, *MUC2*, and *CCL2*, were also consistently upregulated in both TUBB2A KO and TUBB3 KD cell lines compared with control cells. In contrast, TUBB2A KO cells showed coordinated downregulation of osteotropism- and niche-associated transcriptional programs. Specifically, loss of TUBB2A was associated with downregulation of three components of bone-niche integration: (1) loss of the defining osteotropic adhesion molecule *CDH11*, (2) downregulation of Notch and Wnt pathway components (*NRARP*, *NKD1*, *ANKRD6*, and *TCF7L1*), and (3) reduced expression of niche-engaging surface and secreted factors, including *OSCAR*, *CLEC11A*, *MSR1*, and *ENG*. Finally, we observed isotype-specific regulation of melanoma-associated antigen (MAGE) family genes. *MAGEA1* and *MAGEA12* were downregulated in TUBB2A KO cells, whereas *MAGEA6* was upregulated specifically in TUBB3 KD cells. Collectively, the exploratory RNA-seq data suggest that loss or partial depletion of β-tubulin isotypes induces non-redundant transcriptional reprogramming in bone-metastatic breast cancer cells. This reprograming comprised a shared transcriptional signature of inflammatory and matrix-remodeling programs alongside *TUBB2A*- and *TUBB3*-specific downregulation of bone-niche engagement pathways and regulation of cancer–testis antigens. Together, these exploratory findings position β-tubulin isotypes as candidate modulators of tumor–microenvironment interactions.

Among the differentially expressed genes, *IL6* was identified as a prominent candidate and selected for further validation. Consistent with transcriptomic changes, ELISA analysis of conditioned media demonstrated opposing effects. TUBB2A KO cells resulted in a significant increase in IL-6 secretion levels compared with control cells (1.7-fold, **** *p* < 0.0001), whereas TUBB3 KD led to a significant reduction in secreted IL-6 (0.4-fold, **** *p* < 0.0001; Figure 5C). Taken together, these findings indicate that *TUBB2A* and *TUBB3* play distinct and opposing roles in regulating cytokine secretion. The differential effects on IL-6 output are consistent with their non-redundant functions in microtubule organization and vesicle-mediated trafficking.

### 3.6. TUBB2A Knockout and TUBB3 Knockdown Reduce Cell Migration and Invasive Capacity

**Figure 6:**
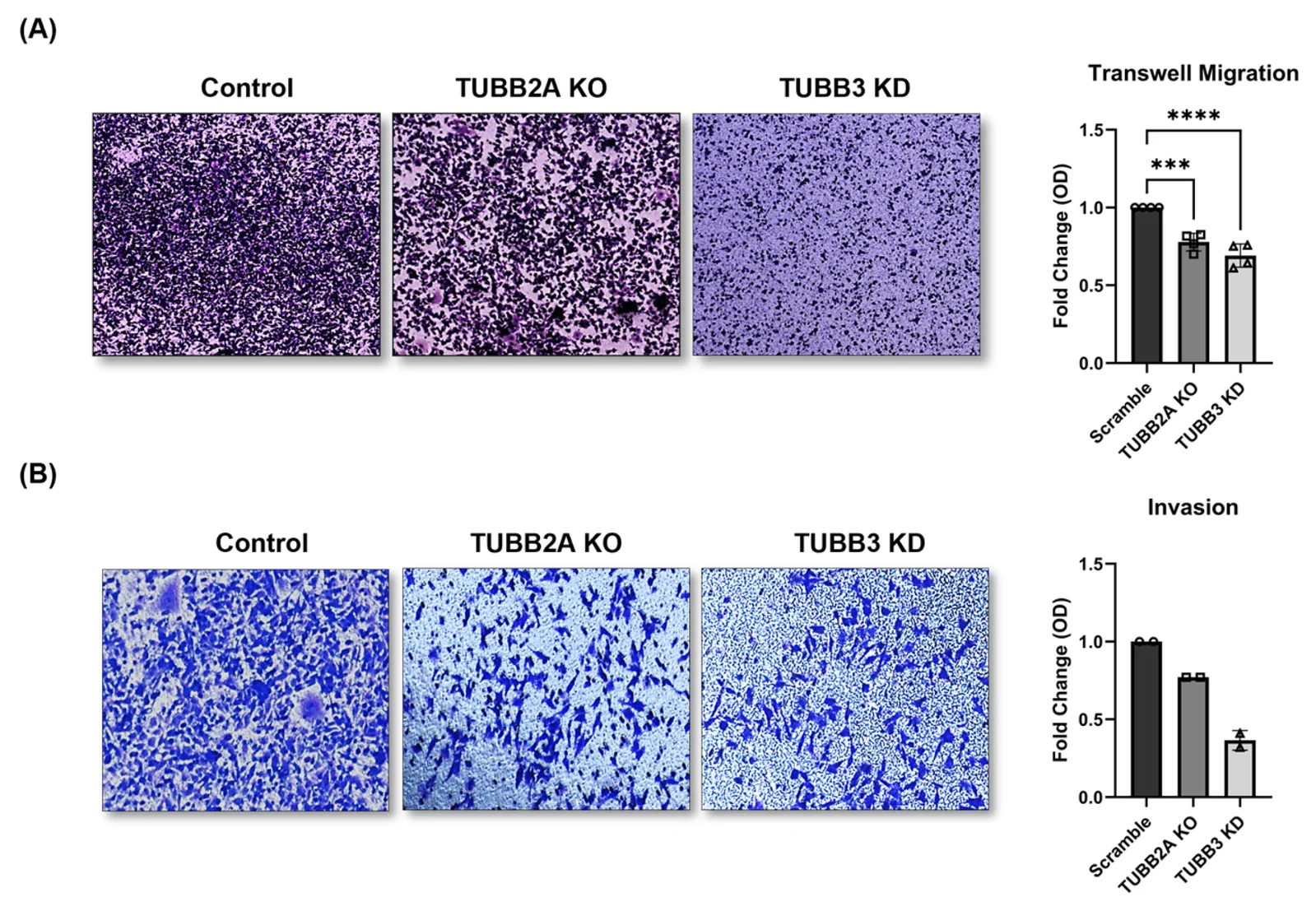
Loss of *TUBB2A* and partial depletion of *TUBB3* alter migration and invasion in bone-metastatic MDA-MB-231 cells. (**A**) Transwell migration assay was performed as follows. Scramble, TUBB2A KO, and TUBB3 KD cells were seeded in 8.0 µm pore transwell inserts and allowed to migrate for 6 h, then fixed and stained with crystal violet. Representative images (left) and quantification of eluted crystal violet by optical density, presented as fold change relative to scramble (right). Bar shading and symbol shape correspond to the cell lines as labeled; each symbol represents one independent experiment. Migration was reduced in both TUBB2A KO (0.78-fold, *** *p* < 0.001) and TUBB3 KD cells (0.69-fold, **** *p* < 0.0001). Bars represent mean ± SD of *n* = 4 independent experiments. One-way ANOVA with Dunnett’s multiple-comparisons test. (**B**) Transwell invasion assay was performed as previously described in (**A**) for 24 h using Matrigel-coated inserts. Invasion was reduced in TUBB2A KO cells (0.77-fold) and more substantially in TUBB3 KD cells (0.37-fold). Bars represent mean ± SD of *n* = 2 independent experiments.

To evaluate the role of *TUBB2A* and *TUBB3* in cell motility, we performed transwell migration assays. Quantitative analysis revealed an approximately 20% decrease in migration in TUBB2A KO cells (*** *p* < 0.001) and a 30% decrease in TUBB3 KD cells (**** *p* < 0.0001) (Figure 6A), indicating a more pronounced requirement for *TUBB3* in migration. We next assessed the invasive capacity of TUBB2A KO and TUBB3 KD cells using Matrigel-coated transwell assays to model extracellular matrix invasion. Consistent with the migration data, both TUBB2A KO and TUBB3 KD cells showed a trend of reduced invasion relative to controls. Specifically, invasion was reduced by approximately 20% in TUBB2A KO cells and 60% in TUBB3 KD cells (Figure 6B). This suggests that reduced *TUBB2A* and *TUBB3* may impair invasion through extracellular matrix components. Together, these findings demonstrate that *TUBB2A* and *TUBB3* are critical regulators of cell migration and likely invasive behavior.

### 3.7. Loss of TUBΒ2A and Partial Depletion of TUBB3 Alter Cellular Adhesion and Focal Adhesion Signaling

**Figure 7:**
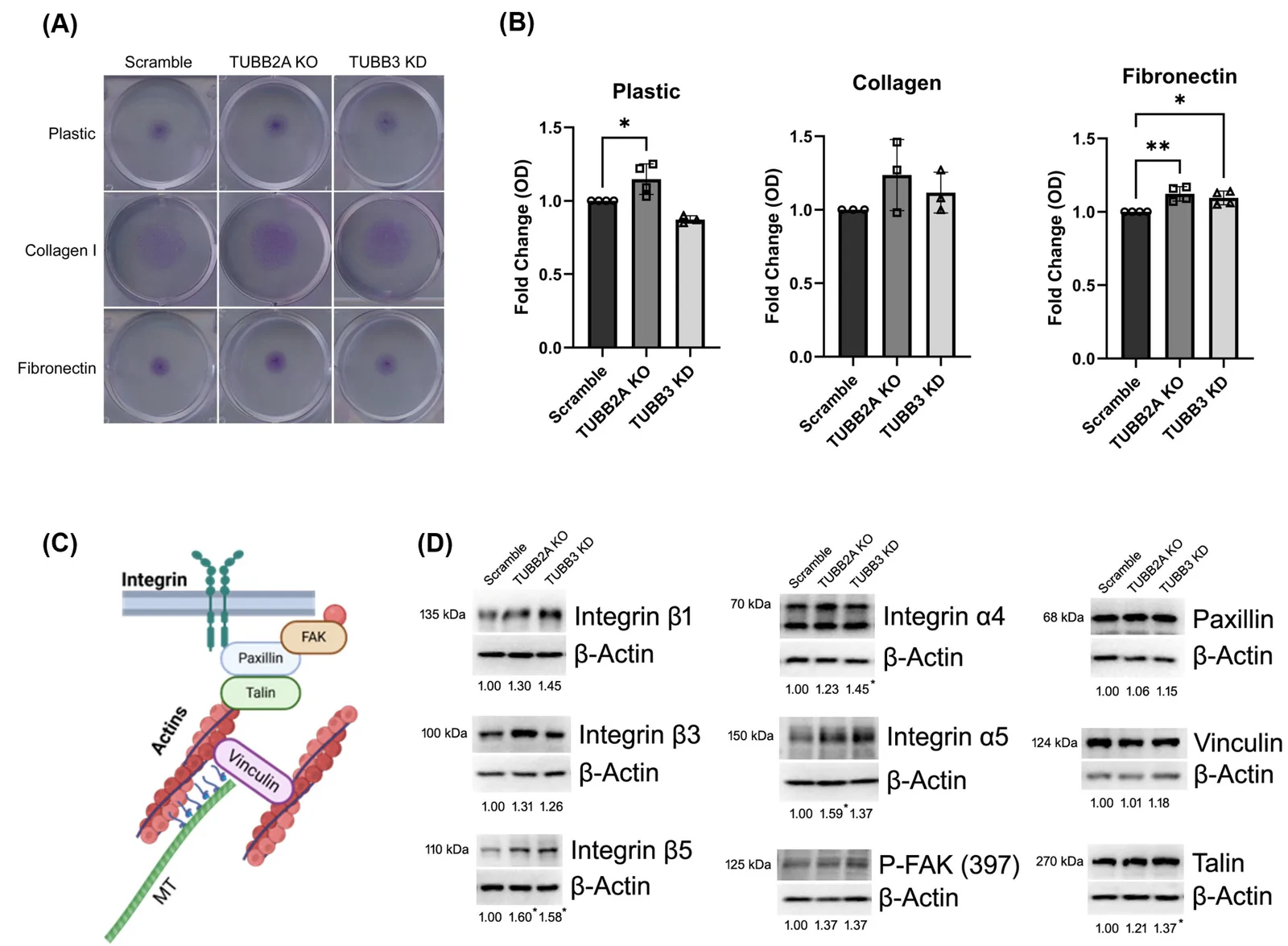
Tubulin-deficient MDA-MB-231 cells exhibit altered substrate adhesion and focal adhesion protein expression. (**A**) Representative scanned image of 12-well plates following crystal violet staining of adherent MDA-MB-231 cells. Cells were seeded as 50,000-cell droplets in 50 µL onto tissue culture plastic, collagen I, or fibronectin. After 2 h, wells were washed to remove non-adherent cells, and remaining adherent cells were stained with crystal violet. (**B**) Quantification of crystal violet staining following solubilization in acetic acid and absorbance measurement at 560 nm. Data are presented as fold change relative to scramble control; symbols represent individual biological samples (*n* = 3–4). Error bars indicate standard deviation. Statistical significance was determined using one-way ANOVA followed by Dunnett’s multiple-comparisons; * *p* < 0.05, ** *p* < 0.01. (**C**) Schematic illustrating interactions among microtubules (MT), actin, focal adhesion complexes, and endosomal trafficking pathways that collectively regulate cell adhesion and signaling; the red circle indicates phosphorylated focal adhesion kinase (FAK), and blue symbols represent MT–actin crosslinking proteins. Loss or reduction of TUBB2A or TUBB3 may disrupt coordination between these networks. (**D**) Western blot analysis of focal adhesion-associated proteins. TUBB2A KO cells showed increased integrin β5 and α5, while TUBB3 KD increased integrin β5, α4, and talin. Quantification represents fold change relative to scramble, with β-actin serving as a loading control. Bars represent mean ± SD of *n* = 3–7 independent sample sets. One-way ANOVA with Dunnett’s multiple-comparisons test; * *p* < 0.05. For original WB Figures please see Supplementary File S1.

To determine whether depletion of *TUBB2A* or *TUBB3* affects cell–matrix adhesion, we performed quantitative cell-adhesion assays. Knockout of *TUBB2A* resulted in a significant increase in cell adhesion on plastic (by approximately 15%), whereas knockdown of *TUBB3* demonstrated a decrease relative to control cells (by approximately 13%; Figure 7A,B), indicating distinct and non-redundant roles for these β-tubulin isotypes in regulating adhesive behavior.

We next assessed substrate-specific adhesion using plates coated with collagen or fibronectin, two major extracellular matrix components. On fibronectin, both TUBB2A KO and TUBB3 KD showed a significant increase in adhesion relative to Scramble control (*p* < 0.01 and *p* < 0.05, respectively; Figure 7A,B). On collagen I, both genotypes trended toward increased adhesion relative to scramble control (TUBB2A KO 1.24-fold, TUBB3 KD 1.12-fold), although this did not reach statistical significance. Because collagen I is the principal organic constituent of bone matrix, adhesion to this substrate is particularly relevant to the engagement of the bone-metastatic niche. The absence of any reduction suggests that loss of TUBB2A or partial depletion of TUBB3 does not impair, and may modestly favor, attachment to bone matrix. While adhesion was enhanced on both substrates compared to uncoated surfaces, fibronectin elicited a significantly greater increase in both TUBB2A KO and TUBB3 KD cells (Figure 7A,B and Supplementary Figure S9). We performed all adhesion assays within a short 2 h time frame, which minimizes the contribution of cell proliferation. Together, these findings suggest that altered MT composition modulates integrin-dependent adhesion, with the strongest effect on fibronectin.

To define molecular changes associated with altered adhesion, we examined the expression of focal adhesion–associated proteins by immunoblotting (Figure 7C,D and Supplementary Figure S10). TUBB2A KO and TUBB3 KD cells displayed increased levels of key focal adhesion components, including integrin β5, integrin β4, integrin α4, integrin α5, and Talin (Figure 7D), all of which are central regulators of focal adhesion assembly and stability. Although knockout of *TUBB2A* increased overall cell adhesion, this was accompanied by reduced migration and invasion, suggesting dysregulated adhesion dynamics rather than enhanced motility. Consistent with this, elevated levels of focal adhesion components, including integrins and Talin, may reflect stabilization of adhesion complexes, which is known to restrain cell migration by limiting adhesion turnover. Collectively, these results indicate that *TUBB2A* and *TUBB3* regulate cell–matrix adhesion by modulating focal adhesion composition and cytoskeletal dynamics.

## 4. Discussion

Breast cancer progression and the development of bone metastasis require coordinated alterations in cytoskeletal organization, intracellular trafficking, and tumor–microenvironment interactions [19,20]. In our study, we identify dysregulation of specific β-tubulin isotypes of MT cytoskeleton as a clinically and biologically relevant feature of aggressive breast cancer. Our data demonstrate that elevated expression of *TUBB2A* and *TUBB3* is associated with reduced relapse-free survival and is enriched in bone-metastatic lesions, highlighting these isotypes as potential drivers of bone metastasis rather than passive structural components of the MT cytoskeleton.

Analysis of a large breast cancer cohort revealed marked functional divergence among closely related β-tubulin isotypes. While increased expression of *TUBB2A*, *TUBB3*, and *TUBB4B* correlated with poor clinical outcomes, *TUBB2B* showed minimal association with relapse-free survival and *TUBB1* was linked to improved prognosis. These findings underscore that changes in β-tubulin composition are not functionally equivalent. The strong association between *TUBB2A* and *TUBB3* expression and adverse patient outcomes supports a model in which selective tubulin isotype usage actively contributes to tumor aggressiveness.

Previous studies have implicated *TUBB3* in promoting migration and metastatic potential, particularly in brain-metastatic breast cancer [20,21,22]. Our findings extend these observations by demonstrating overall enrichment of tubulin-β2-positive cells and moderate to strong tubulin-β3-positive cells in bone metastases, suggesting that these isotypes facilitate adaptation of breast cancer cells to the unique bone microenvironment and/or have a specific role in tumor cell interaction with the microenvironment. The cohort utilized in our study was enriched for HR+/HER2− tumors, and many patients had received taxane-based therapies and bone-targeted agents (Supplementary Table S1), both of which may influence β-tubulin expression and confound interpretation. Validation in larger, more clinically diverse cohorts may further confirm the generalizability of these findings. Future in vivo studies will further define the specific functions of these isotypes in tumor cell survival and adaptation in bone. The bone microenvironment niche is characterized by a rigid, mineralized extracellular matrix, high mechanical stress, and extensive crosstalk with stromal and immune cells [23]. The selective abundance of tubulin β2 and tubulin β3 in bone lesions relative to matched primary tumors indicates that these isotypes may be required to support both survival and expansion following metastatic seeding. Normal mammary tissue exhibits higher tubulin β2 staining relative to tubulin β3; however, this relationship is reversed in primary tumors and further amplified in bone metastases. Such reprogramming of tubulin composition likely alters MT dynamics in ways that favor metastatic behavior. Consistent with this notion, depletion of either *TUBB2A* or *TUBB3* impaired MT regrowth following depolymerization and altered the proportion of cells at all phases of mitosis [24,25]. Given the proliferative constraints imposed by the bone niche, enhanced expression of these isotypes may confer a selective advantage by supporting the MT dynamics required for efficient mitosis and colony outgrowth. Our findings align with prior reports showing critical roles for β-tubulin isotypes in mitotic spindle function [26]. Overexpression of mutant *TUBB2A* disrupts spindle bipolarity and mitotic progression [27]. *TUBB3* expression is likewise tightly coupled to the cell cycle, peaking at the G2/M transition [28,29,30], and localizing to the mitotic spindle across cell types [31]. Here we demonstrated knockdown of *TUBB3* resulted in a significant reduction in G2/M-phase cells, reinforcing the idea that *TUBB3* is integral to cell-cycle control. Although depletion of TUBB2A and TUBB3 impaired microtubule regrowth, future studies employing live-cell imaging and direct measurements of microtubule polymerization will provide deeper insight into their specific roles in microtubule assembly, stability, and remodeling.

Beyond cell-cycle regulation, our results uncover a previously underappreciated role for β-tubulin isotypes in intracellular trafficking and cytokine signaling. Loss of *TUBB2A* and partial depletion of *TUBB3* disrupted endosomal organization, resulting in enlarged Rab5- and increase in Caveolin-1-positive vesicles, indicative of defective endocytic subcellular distribution. Efficient vesicular transport is critical for receptor recycling, signal transduction, and secretion of factors that mediate tumor–bone crosstalk [32,33,34,35]. Notably, *TUBB2A* and *TUBB3* exerted opposing effects on IL-6 secretion, *TUBB2A* depletion increased IL-6 output, whereas *TUBB3* knockdown reduced it. Given the central role of IL-6 in osteoclast activation and the osteolytic “vicious cycle” of bone metastasis [36,37], these findings support a model in which β-tubulin isotypes fine-tune inflammatory signaling through microtubule-dependent trafficking pathways. Differential interactions of isotype-specific C-terminal tails with kinesin and dynein motors may underlie these effects by modulating cargo transport efficiency [38].

Our data further indicates that *TUBB2A* and *TUBB3* contribute to cell–matrix interactions and invasive behavior. Depletion of either isotype resulted in increased adhesion to fibronectin and reduced migratory capacity, with a consistent trend toward reduced invasion. These phenotypes are consistent with impaired cytoskeletal dynamics, as efficient migration and invasion require coordinated focal adhesion turnover to enable cell detachment and forward movement through dense extracellular matrices such as bone [39]. Consistent with this, we observed alterations in focal adhesion-associated proteins in TUBB2A KO and TUBB3 KD cells, suggesting that these β-tubulin isotypes contribute to the regulation of adhesion dynamics. Disruption of this balance likely stabilizes cell–substrate interactions, thereby limiting motility and invasive potential. These findings provide a mechanistic basis for the enrichment of tubulin β2 and tubulin β3 in bone metastases, where efficient remodeling of cell–matrix contacts is critical for tumor cell dissemination and colonization. This effect appears not to be readily compensated by other β-tubulin isotypes, highlighting a degree of functional specificity within the tubulin family in regulating tumor cell plasticity. A limitation of the current study is the absence of a complex bone microenvironment. Therefore, future studies using bone-specific extracellular matrix components and osteoblast–osteoclast co-culture models will be necessary to validate the impact of TUBB2A and TUBB3 knockouts and rescue experiments by altering downstream pathways on tumor cell adhesion, migration, and metastatic behavior under physiologically relevant conditions.

Metastatic breast cancer progression relies on coordinated cytoskeletal remodeling to integrate inflammatory signaling with interactions in distant tissue niches, particularly bone. Our exploratory RNA-Seq data suggest β-tubulin isotypes as candidate regulators of metastatic transcriptional programs. Loss of *TUBB2A* was associated with a transcriptional signature of NF-κB/IRF/AP-1 inflammatory networks, including increased expression of osteoclastogenic cytokines (*IL-1β*, *IL-6*) and chemokines (*CSF3*, *CCL2*, *CXCR2*). These mediators are established drivers of osteoclast recruitment and activation, promoting bone resorption and release of matrix-sequestered growth factors such as TGF-β, IGFs, and BMPs that fuel metastatic expansion within the bone microenvironment [37,40]. Such inflammatory reprogramming suggests that TUBB2A deficiency may enhance the ability of tumor cells to initiate osteolytic remodeling and create a permissive pre-metastatic niche [41].

Interestingly, despite this pro-inflammatory phenotype, TUBB2A loss simultaneously downregulated several gene programs associated with stable bone colonization and niche adaptation. Among the most notable changes was the downregulation of CDH11, an osteotropic adhesion molecule that facilitates interactions between breast cancer cells and osteoblasts and has been linked to preferential skeletal metastasis. TUBB2A-deficient cells also exhibited coordinated downregulation of Notch and Wnt pathway components, which regulate osteomimicry, stemness, tumor dormancy, and communication with bone-resident stromal cells. Notch signaling promotes reciprocal interactions between tumor cells, osteoblasts, and osteoclast precursors, whereas Wnt pathway activity supports skeletal colonization and metastatic persistence [42,43,44]. These findings suggest that *TUBB2A* deficiency favors inflammatory niche destabilization rather than stable bone residency. In contrast, *TUBB3* knockdown was associated with increased expression of melanoma-associated antigen (MAGE) family genes, including *MAGEA6*, a pattern consistent with prior reports linking MAGE expression to metastatic competence, stress adaptation, and immune modulation in advanced cancers [45,46,47]. Consistent with this, TUBB3-deficient cells showed transcriptional enrichment of pathways linked to extracellular matrix remodeling and cellular adaptation, suggesting that TUBB3 may normally restrain programs supporting niche survival and metastatic plasticity. Together, these findings suggest that β-tubulin isotypes differentially regulate tumor-bone microenvironment interactions and point to complementary roles across stages of bone metastasis, from early niche conditioning to long-term metastatic colonization.

While our study has some limitations, we observed variability in the efficiency of *TUBB3* knockdown via CRISPR/Cas9 across passages, likely reflecting the essential role of *TUBB3* in mitotic spindle formation and cellular viability. Our in vitro analyses utilized a well-established bone-tropic breast cancer MDA-MB-231 model, enabling detailed mechanistic insight. Importantly, these findings were complemented with analyses of patient tumor samples, unbiased transcriptional profiling, and publicly available breast cancer datasets. We acknowledge that the clinical cohort size is relatively limited and that patient-specific factors may influence tubulin isotype expression levels. Furthermore, multiple α and β-tubulin isotypes contribute to cytoskeletal remodeling and metastatic progression [48]; however, our data identified TUBB2A and TUBB3 as the most robust and functionally significant candidates in this context. Together, these integrated approaches strengthen our conclusions and highlight a contributory role for both *TUBB2A* and *TUBB3* in promoting metastatic properties of breast cancer cells. Although our findings support a role for TUBB2A and TUBB3 in breast cancer bone metastasis, validation in patient-derived models and preclinical mouse models are needed to establish their functional importance and therapeutic potential in driving metastatic progression and tumor growth in bone.

Collectively, our findings support a model in which the selective enrichment of TUBB2A-positive cells and moderate-to-strong TUBB3-expressing breast cancer cells enhances metastatic properties. These tubulins isotypes appear to facilitate key processes required for metastatic progression, including mitotic progression, vesicular trafficking, cytokine secretion, and the dynamic regulation of cell–matrix interactions. The strong association of elevated TUBB2A and TUBB3 expression with poor patient outcomes further underscores their potential value as prognostic biomarkers. More broadly, our data identify β-tubulin isotype composition as a determinant of metastatic competence on bone-metastatic breast cancer cells. Future studies are warranted to determine whether selective targeting of isotype-specific microtubule functions can disrupt tumor-bone interactions while avoiding the broad cytotoxic effects associated with conventional microtubule-targeting agents [49]. Importantly, growing evidence indicates that individual tubulin isotypes are not functionally redundant but instead exert distinct effects on microtubule dynamics, intracellular trafficking, signaling pathways, and cellular behavior in a context-dependent manner [50]. This functional specialization suggests that disease-associated tubulin isotypes may play disproportionately important roles in pathological processes while being less critical for normal tissue homeostasis. Furthermore, tubulin isotype expression is often cell-type- and differentiation-stage-specific [51]. These findings raise the possibility that isoform-selective targeting or tissue-specific delivery approaches could improve therapeutic precision and minimize systemic toxicities, including neurotoxicity that has historically limited microtubule-targeting therapies [52]. Together, these findings highlight tubulin isotype-specific functions as potential regulators of bone metastasis and promising therapeutic targets.

## 5. Conclusions

Our findings demonstrate that TUBB2A and TUBB3 are key regulators of metastatic behavior in bone-metastatic breast cancer. Elevated expression of these β-tubulin isotypes is associated with poor patient outcomes and increased abundance in bone-metastatic lesions. Functional reduction in either TUBB2A or TUBB3 disrupted microtubule dynamics, cell-cycle progression, endosomal trafficking, migration, and invasion while enhancing cell adhesion. In addition, transcriptomic analyses linked these changes to cytokine and inflammatory signaling pathways, highlighting a role for these isotypes in tumor–microenvironment interactions. Collectively, these results identify TUBB2A and TUBB3 as important contributors of metastatic phenotypes and support their potential utility as biomarkers and therapeutic targets in breast cancer bone metastasis.

## Data Availability

The data that supports the findings of this study are available from the corresponding author upon reasonable request.

## Supplementary Materials

The following supporting information can be downloaded at: https://www.mdpi.com/article/10.3390/cancers18213418/s1, Figure S1: Clinical impact of microtubule isotypes in breast cancer; Figure S2: α-tubulin isotype differential expression in breast cancer; Figure S3: TUBB2A KO and TUBB3 KD validation by NGS; Figure S4: TUBB3 in mitotic spindle and cell morphology; Figure S5: Additional isotype-specific compensation; Figure S6: Full microtubule regrowth time course; Figure S7: Binning Rab5-puncta size; Figure S8: Gene ontology enrichment analysis of TUBB2A KO and TUBB3 KD cells; Figure S9: Corresponding brightfield images of adhesion assay; Figure S10: Focal adhesion protein expression in tubulin-deficient cells; Table S1: Clinicopathologic characteristics of the matched primary/bone-metastatic cohort (*n* = 16); Supplementary File S1—Original WB Figures.
