# Supplementary material for: A Novel Function of Tubulin β2a and Tubulin β3 in Regulating Metastatic Properties in Bone-Metastatic Breast Cancer Cells
Source: Cancers (Basel). 2026 Oct 22;18(21):3418. doi: 10.3390/cancers18213418 (PMC13761978; doi:10.3390/cancers18213418)

Full Western Blots Supplementary

The top row (**Main Figure**) shows the cropped blots as presented in the manuscript. The middle row (**Expanded Main Figure**) shows the full, uncropped overlay image for both the target protein and its corresponding β-actin loading control. The overlay is a composite of the membrane photograph with the chemiluminescent TIFF superimposed. It is included here to display the molecular weight ladder, not detectable on the chemiluminescent capture. Quantification was performed on the original chemiluminescent TIFF image shown in the main figure. Densitometry values are therefore not reproducible from the overlay, and for some blots the overlay signal appears weaker than in the original TIFF. Values directly beneath each expanded blot represent densitometry measurements for the respective target bands, and values in parentheses represent measurements of the corresponding β-actin bands. Lanes are labeled 1 = Scramble, 2 = TUBB2A KO, and 3 = TUBB3 KD, indicated by dashed lines and numbering above each expanded panel. Arrows denote the probed target band. No manual image adjustments were applied to any image. Differences in brightness, contrast, or background between panels result from automatic per-image scaling by the iBright imaging system together with the different primary antibodies and imaging durations used for each target. The bottom row (**Bar Graphs of Averaged Sample Sets**) shows target protein signal normalized to β-actin and then to Scramble, expressed as fold change. Bars represent mean ± SD with individual replicates overlaid.

Supplementary Figure: Full Western Blots – Microtubule Isotypes

Main Figure

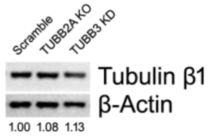

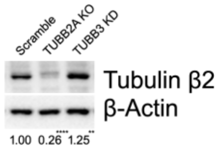

Expanded Main Figure

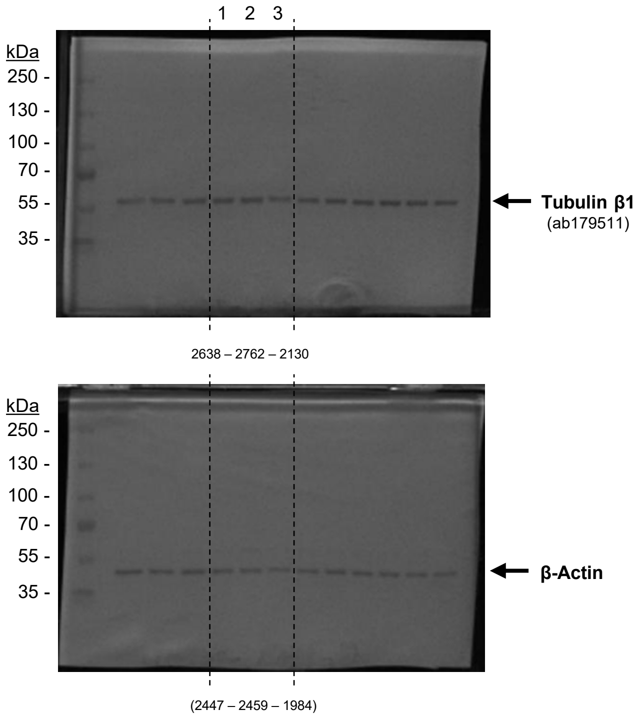

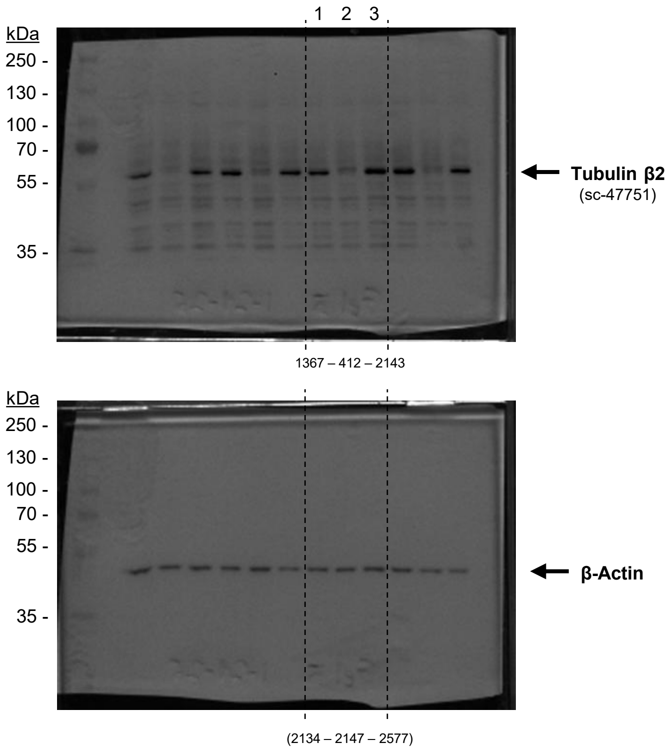

Bar Graphs of Averaged Sample Sets

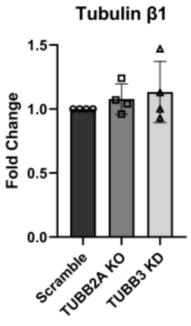

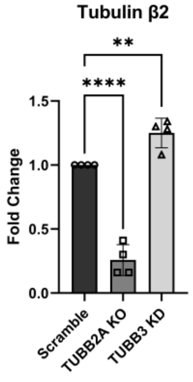

Supplementary Figure: Full Western Blots – Microtubule Isotypes

Main Figure

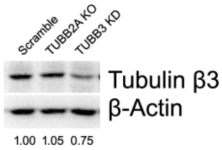

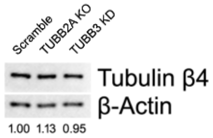

Expanded Main Figure

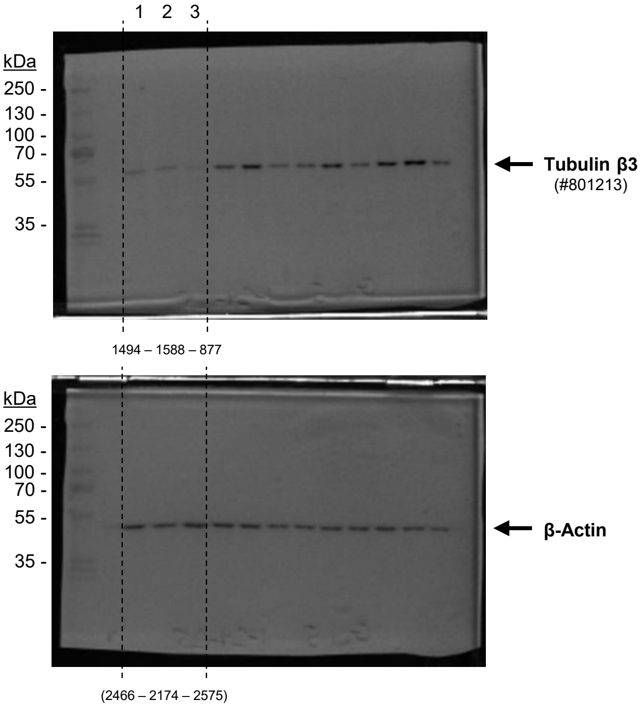

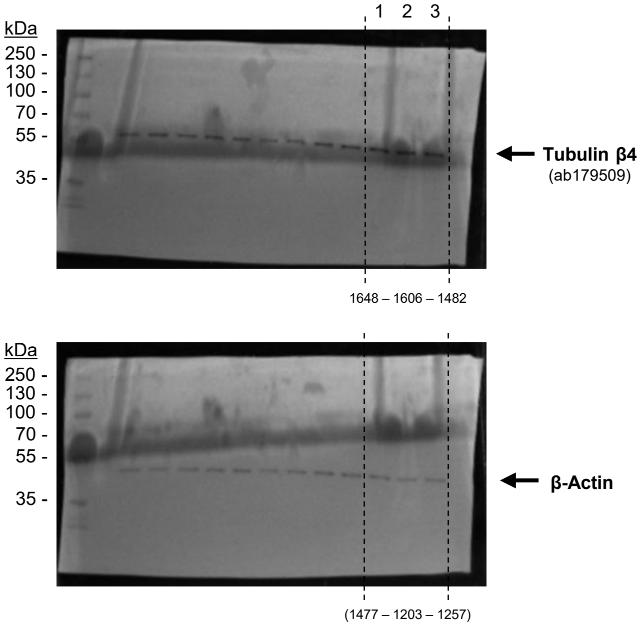

Bar Graphs of Averaged Sample Sets

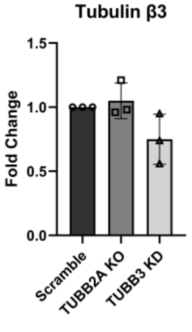

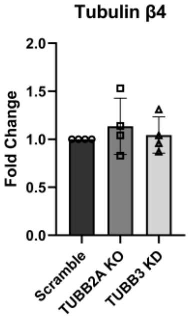

Supplementary Figure: Full Western Blots – Microtubule Isotypes

Main Figure

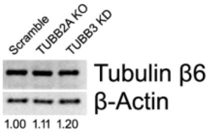

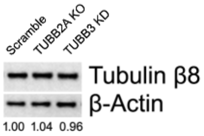

Expanded Main Figure

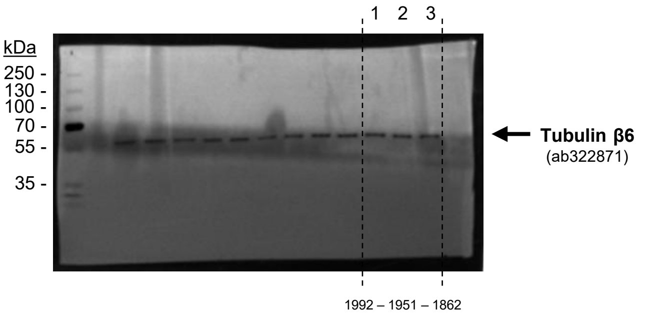

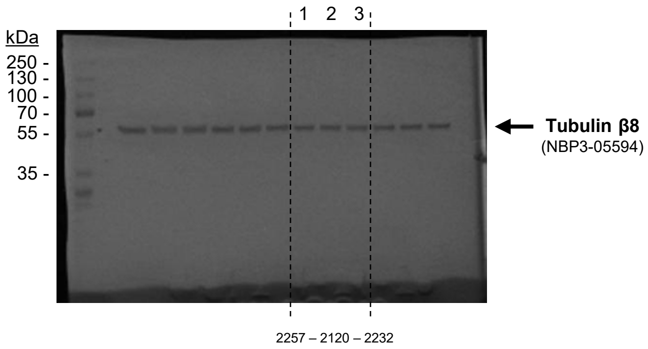

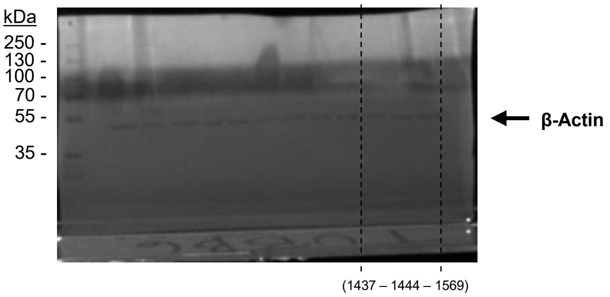

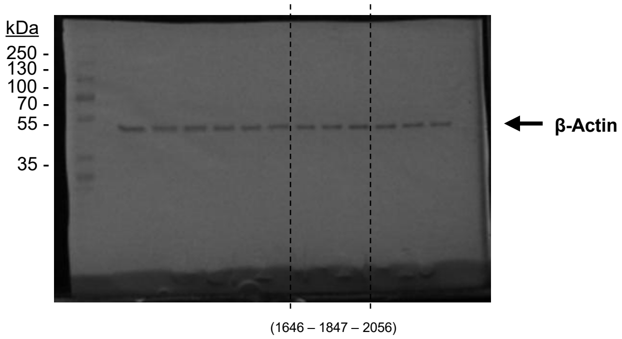

Bar Graphs of Averaged Sample Sets

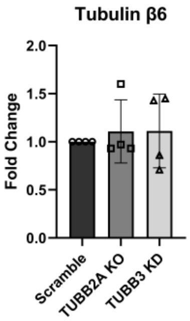

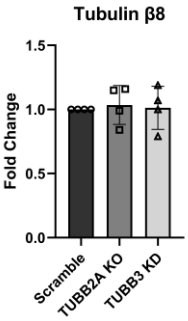

Supplementary Figure: Full Western Blots – Microtubule Isotypes

Main Figure

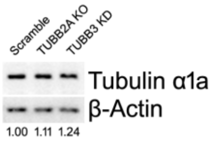

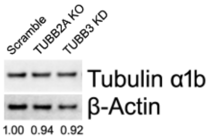

Expanded Main Figure

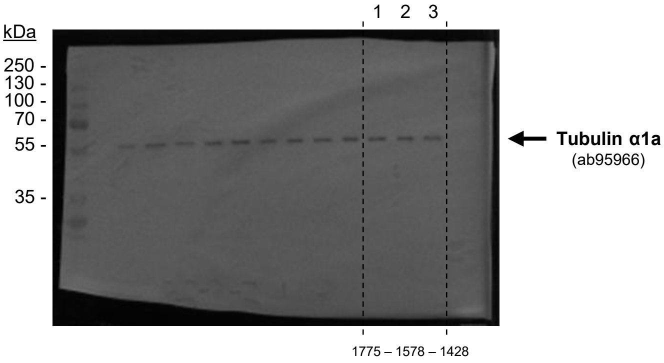

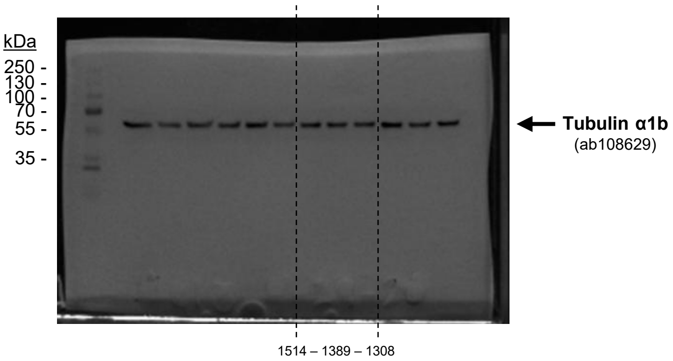

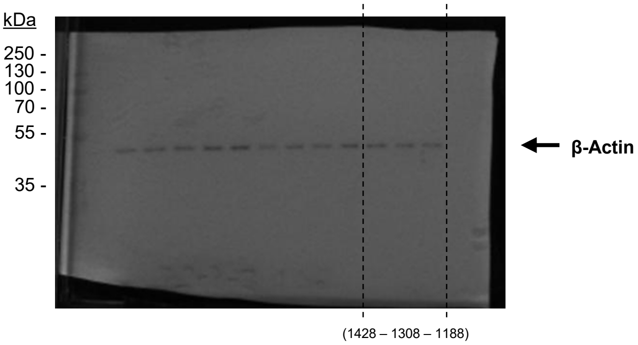

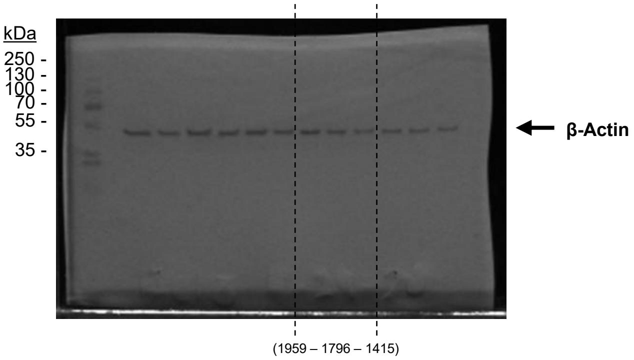

Bar Graphs of Averaged Sample Sets

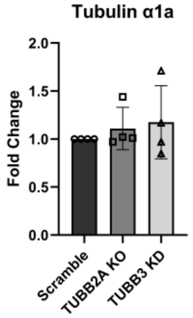

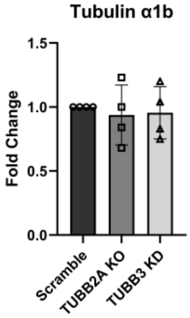

Supplementary Figure: Full Western Blots – Microtubule Isotypes

Main Figure

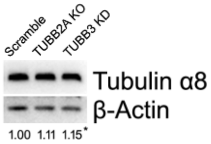

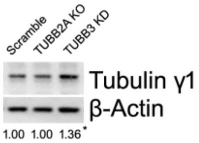

Expanded Main Figure

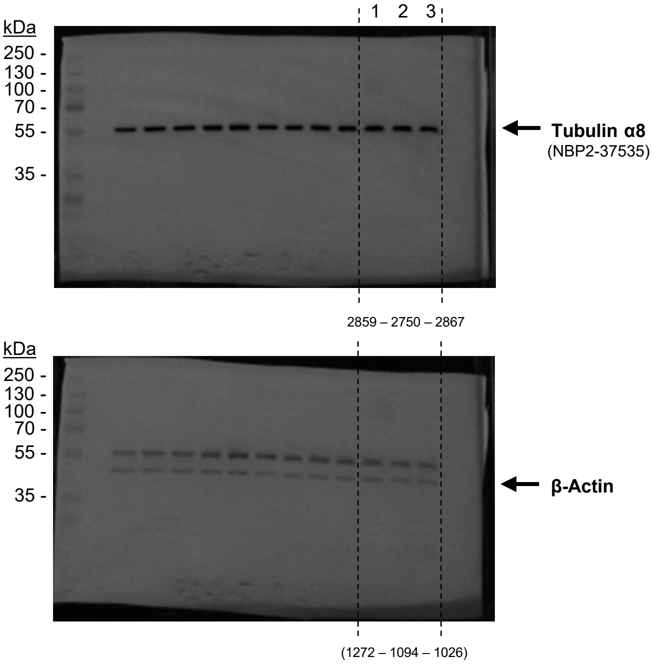

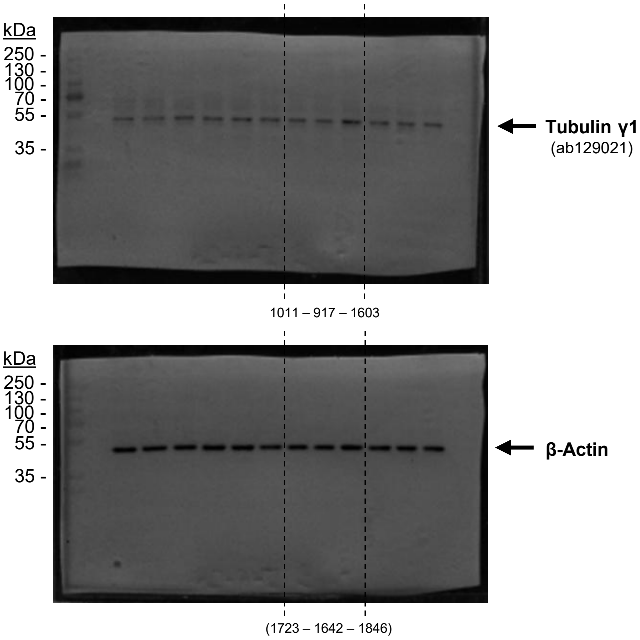

Bar Graphs of Averaged Sample Sets

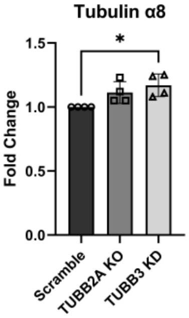

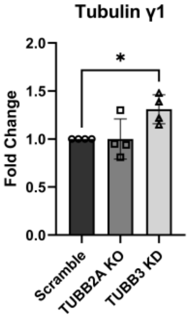

Supplementary Figure: Full Western Blots – Focal Adhesion Proteins

Main Figure

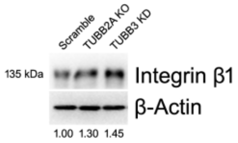

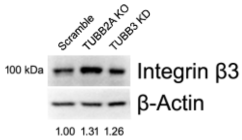

Expanded Main Figure

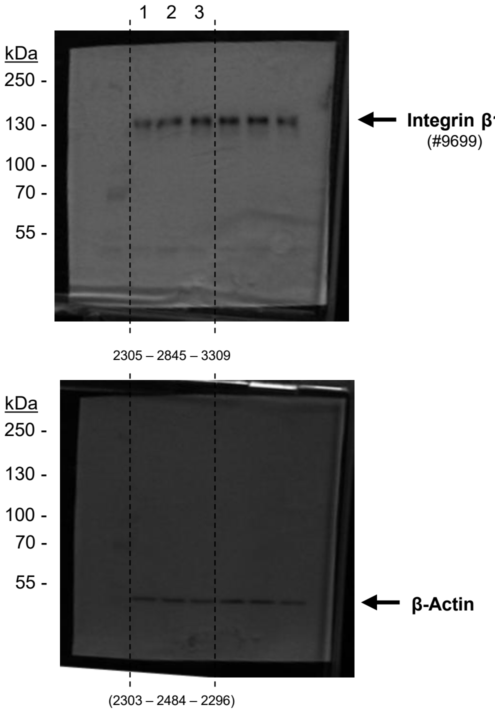

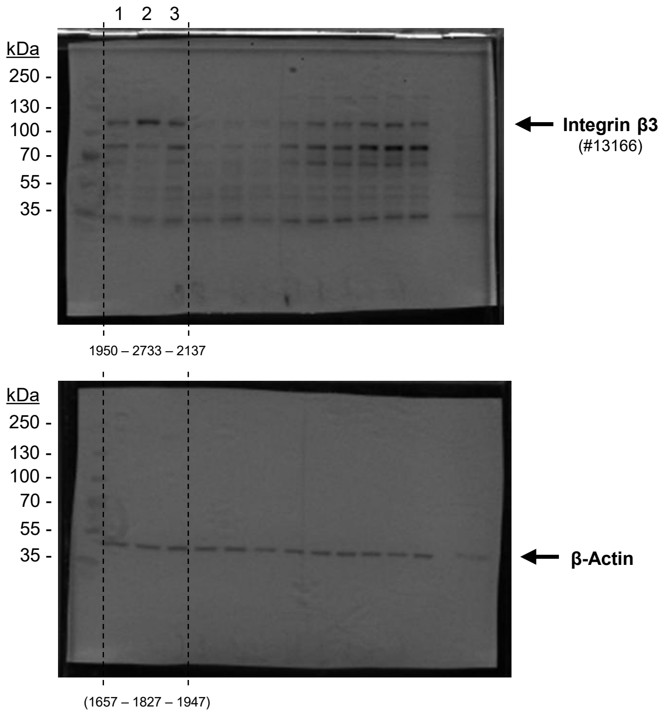

Bar Graphs of Averaged Sample Sets

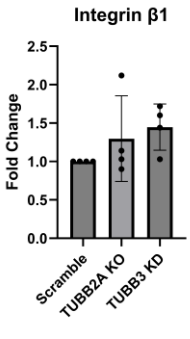

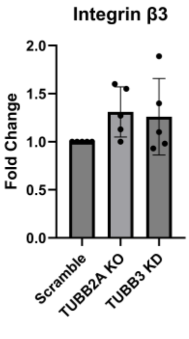

Supplementary Figure: Full Western Blots – Focal Adhesion Proteins

Main Figure

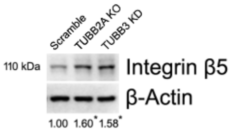

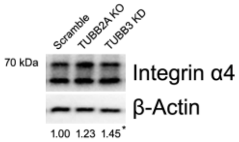

Expanded Main Figure

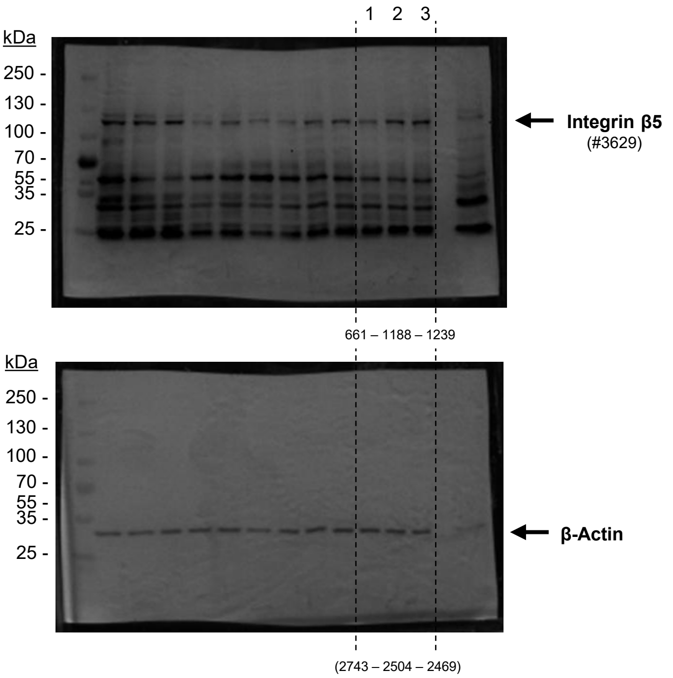

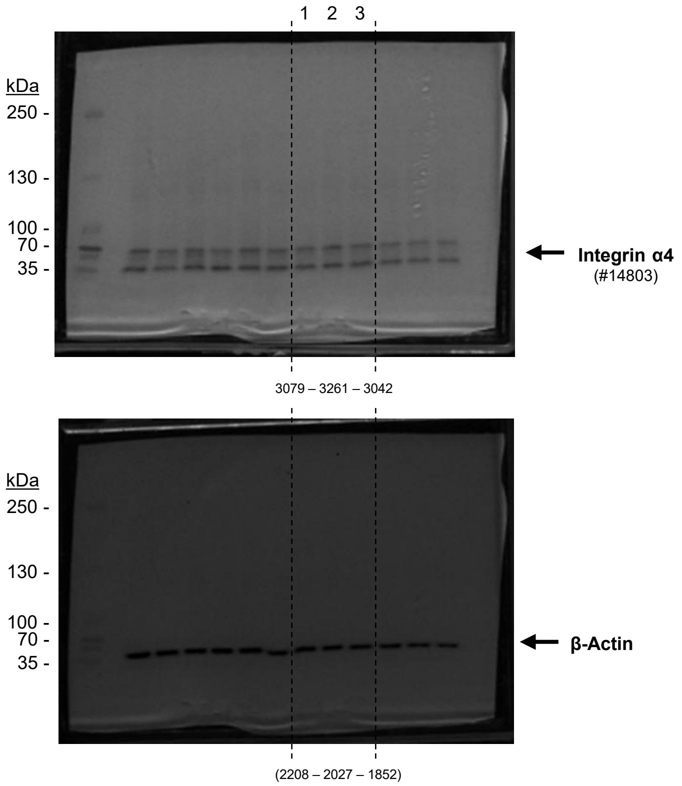

Bar Graphs of Averaged Sample Sets

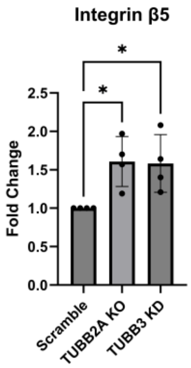

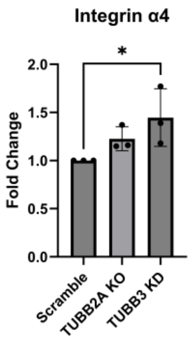

Supplementary Figure: Full Western Blots – Focal Adhesion Proteins

Main Figure

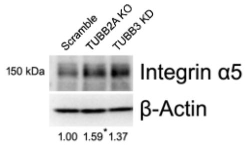

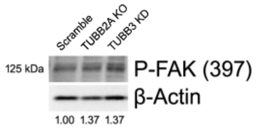

Expanded Main Figure

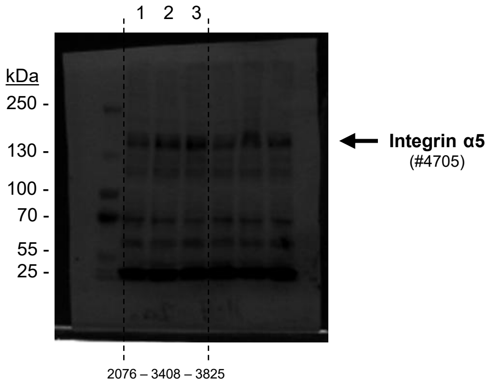

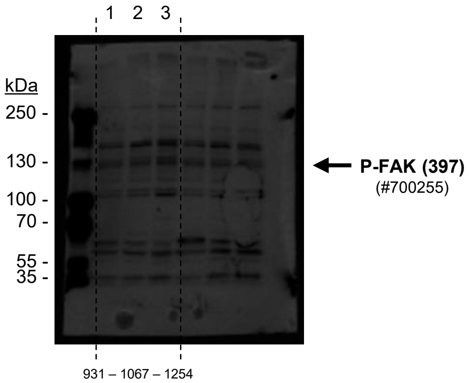

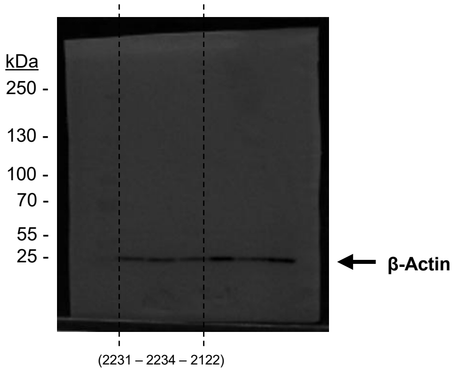

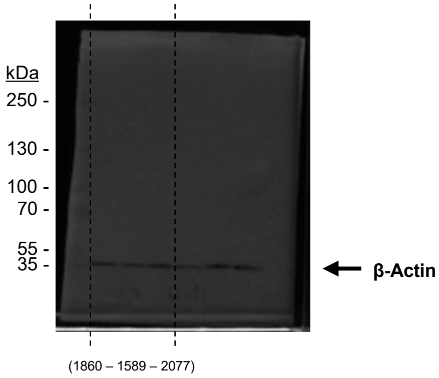

Bar Graphs of Averaged Sample Sets

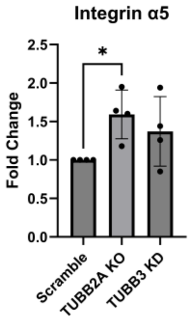

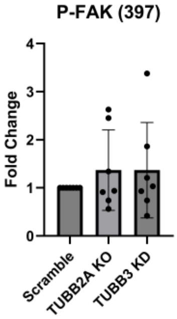

Supplementary Figure: Full Western Blots – Focal Adhesion Proteins

Main Figure

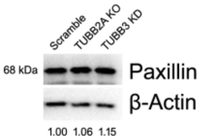

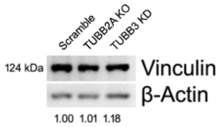

Expanded Main Figure

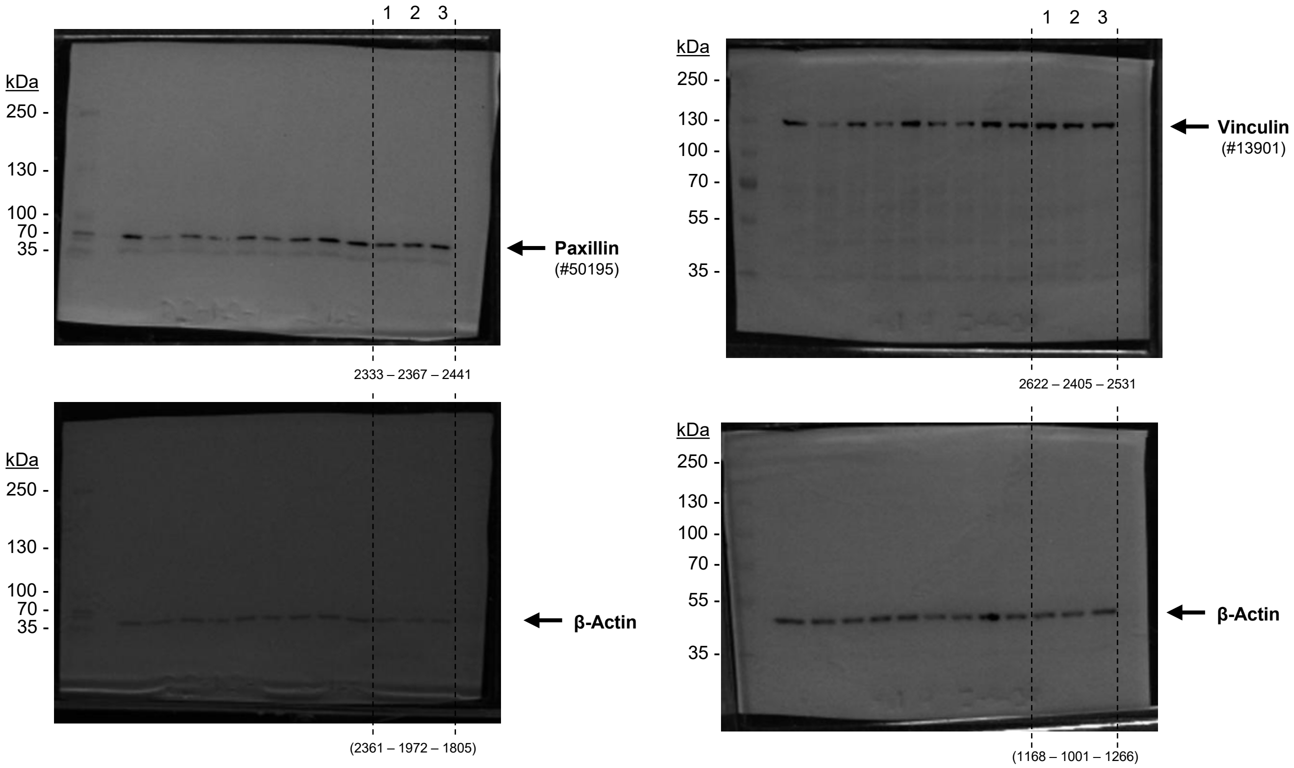

Bar Graphs of Averaged Sample Sets

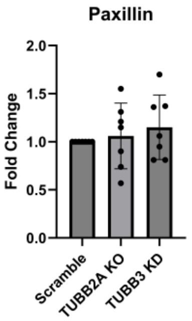

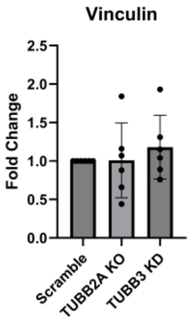

Supplementary Figure: Full Western Blots – Focal Adhesion Proteins

Main Figure

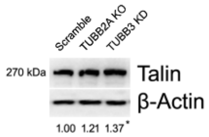

Expanded Main Figure

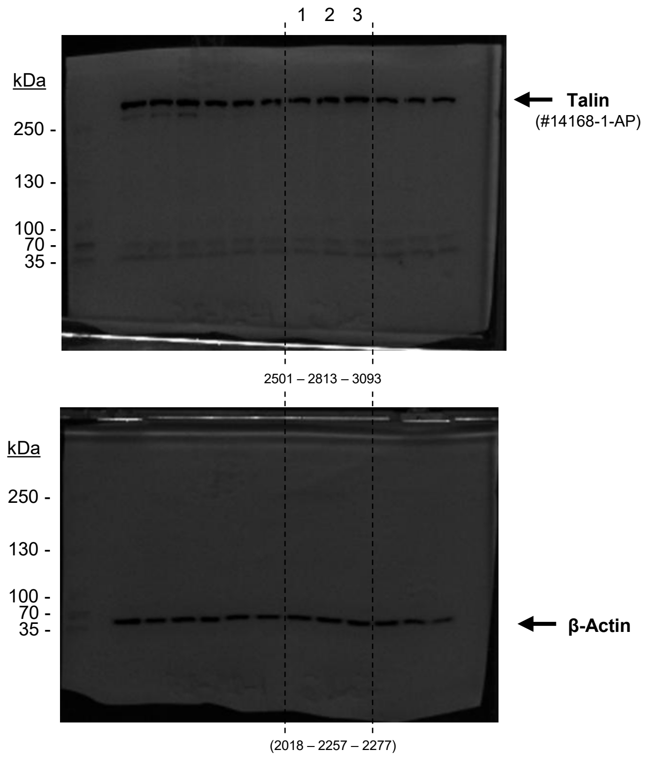

Bar Graphs of Averaged Sample Sets

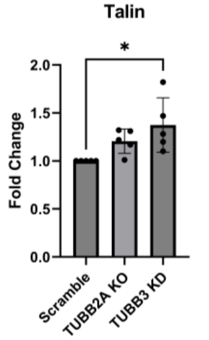

Supplement: Supplementary file 1 [file cancers-18-03418-s001.zip › cancers-4496023-File S1-Original WB Figures.pdf]
