# Supplementary material for: A Novel Function of Tubulin β2a and Tubulin β3 in Regulating Metastatic Properties in Bone-Metastatic Breast Cancer Cells
Source: Cancers (Basel). 2026 Oct 22;18(21):3418. doi: 10.3390/cancers18213418 (PMC13761978; doi:10.3390/cancers18213418)
Supplement: Supplementary file 1 [file cancers-18-03418-s001.zip › cancers-4496023-Supplementary Figures.pdf]

Supplemental Figure S1: Clinical Impact of Microtubule Isotypes in Breast Cancer

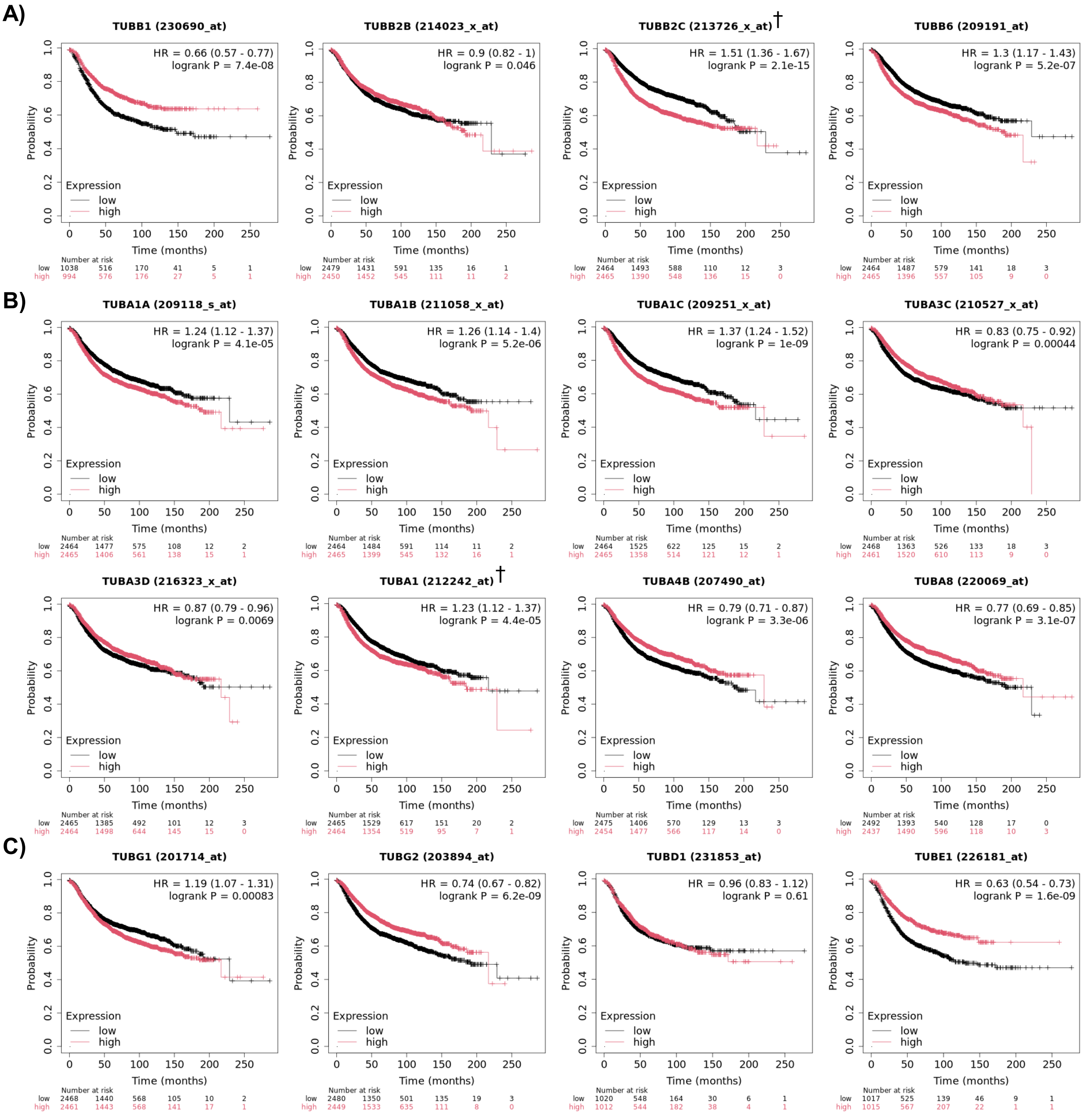

Supplemental Figure S1: Clinical Impact of Microtubule Isotypes in Breast Cancer. Kaplan-Meier plots correlating gene-chip mRNA expression of tubulin isotypes to the survival of breast cancer patients (n = 4929). Plots were generated using KM Plotter (https://kmplot.com/analysis/), including all breast cancer subtypes without restriction by treatment, with patients stratified at the median. Panels are titled with the gene symbol as reported by KM Plotter; † denotes genes annotated under a retired symbol, for which the current symbol is used throughout the text: *TUBB2C* = *TUBB4B* and *TUBA1* = *TUBA4A*. *TUBB2A* and *TUBB3* are presented in Figure 1A. **A)** β-tubulin isotypes. High expression of *TUBB4B* (HR 1.51) and *TUBB6* (HR 1.30) was associated with worse survival, whereas high expression of *TUBB1* (HR 0.66) and *TUBB2B* (HR 0.90) was associated with improved survival. **B)** α-tubulin isotypes. High expression of *TUBA1A* (HR 1.24), *TUBA1B* (HR 1.26), *TUBA1C* (HR 1.37), and *TUBA4A* (HR 1.23) was associated with worse survival, whereas high expression of *TUBA3C* (HR 0.83), *TUBA3D* (HR 0.87), *TUBA4B* (HR 0.79), and *TUBA8* (HR 0.77) was associated with improved survival. **C)** γ-, δ-, and ε-tubulins. High *TUBG1* expression (HR 1.19) was associated with worse survival, while high *TUBG2* (HR 0.74) and *TUBE1* (HR 0.63) were associated with improved survival. *TUBD1* showed no significant association with survival. Unlike the α- and β-tubulin isotypes, γ-tubulin functions in microtubule nucleation and δ- and ε-tubulins are centriole-associated rather than components of the microtubule lattice.

Supplemental Figure S2: α-tubulin Isotype Differential Expression in Breast Cancer

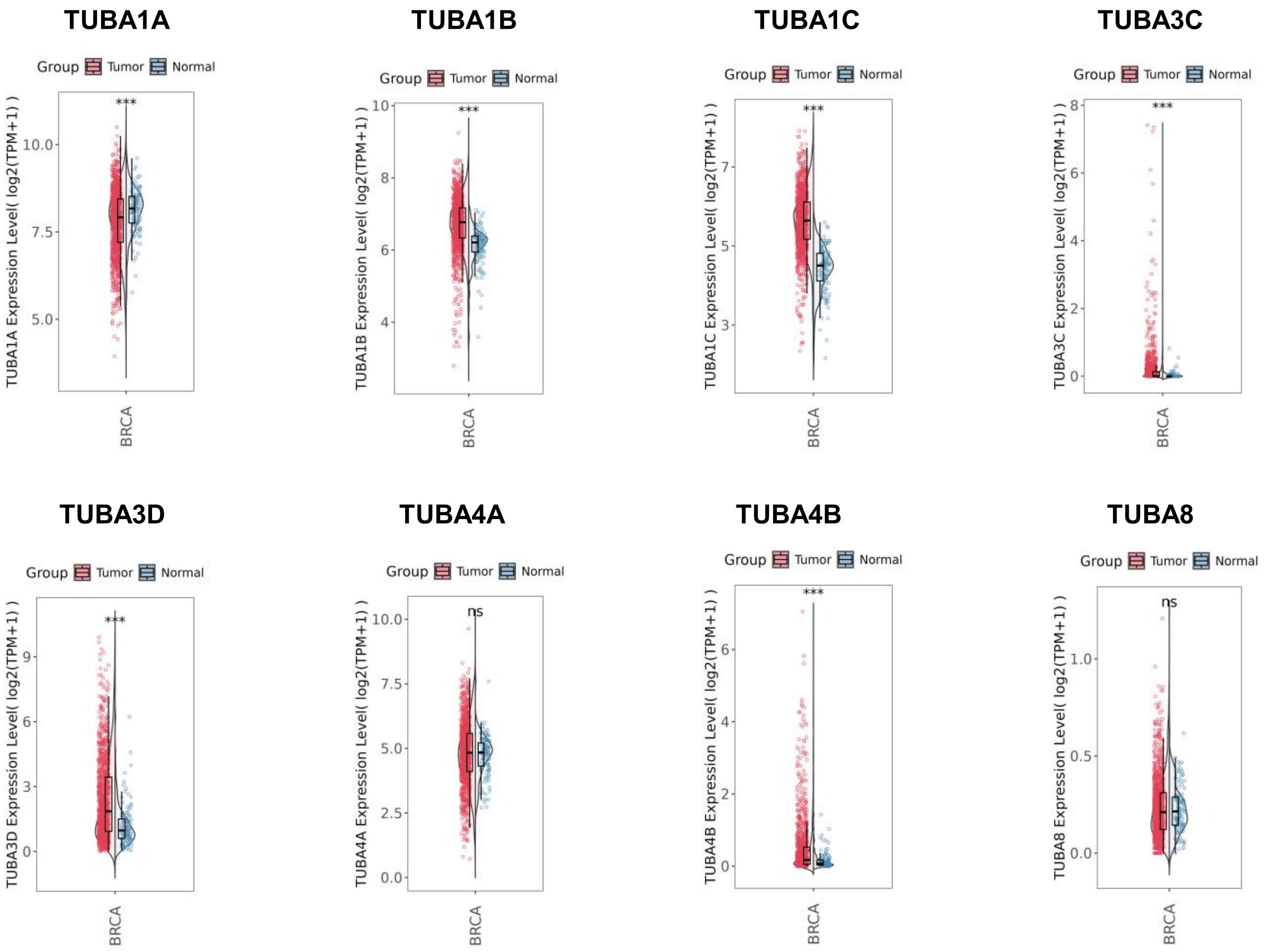

Supplementary Figure S2: α-tubulin Isotype Differential Expression in Breast Cancer. Expression of *TUBA1A*, *TUBA1B*, *TUBA1C*, *TUBA3C*, *TUBA3D*, *TUBA4A*, *TUBA4B*, and *TUBA8* (log_2[TPM+1]) in BRCA tumor (red) versus adjacent normal (blue) breast tissue from TCGA, analyzed using TIMER2.0 (http://timer.cistrome.org/). *TUBA1B*, *TUBA1C*, *TUBA3C*, *TUBA3D*, and *TUBA4B* were significantly elevated in tumor tissue relative to normal, whereas *TUBA1A* was significantly reduced. *TUBA4A* and *TUBA8* showed no significant difference (n=1,093 tumor, n=112 normal; ***p<0.001; ns, not significant). TPM, transcripts per million. Collectively, these findings reveal coordinated upregulation of multiple α-tubulin isotypes in BRCA, consistent with cytoskeletal remodeling in tumorigenesis.

Supplementary Figure S3: TUBB2A KO and TUBB3 KD Validation by NGS

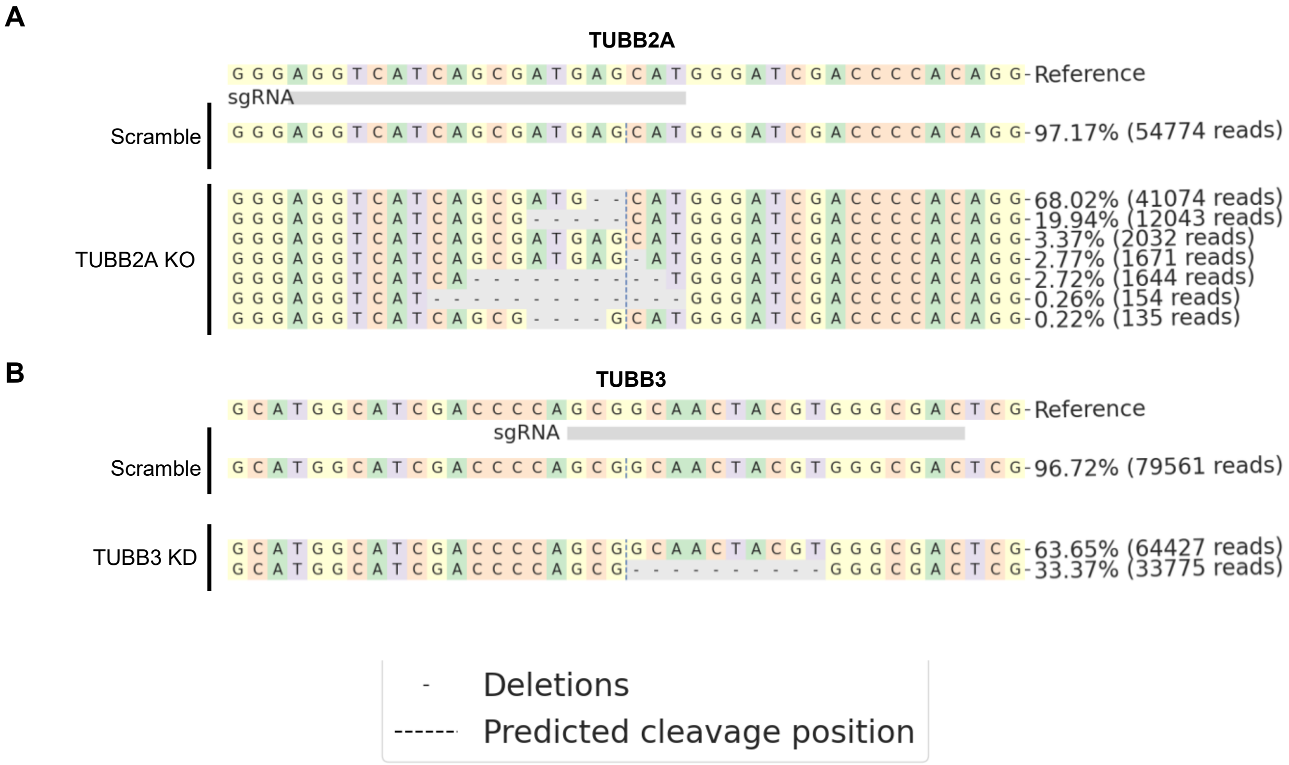

Supplementary Figure S3: TUBB2A KO and TUBB3 KD Validation by NGS. Genomic DNA was isolated from single-cell derived clones and PCR amplified across the target site. Amplicons were subjected to next-generation sequencing and analyzed with CRISPResso2. Frequency plots show the distribution of sequence reads for each clone. The guide RNA target sequence (gray bar) and predicted Cas9 cleavage site (dashed line) are indicated. **A)** Allele frequency distribution at the *TUBB2A* target site in the TUBB2A KO clone. 96.6% of reads carried an indel, with 3.4% matching the unmodified reference sequence. The predominant alleles were a 2 bp deletion (68% of reads) and a 5 bp deletion (20%), both predicted to cause a frameshift; the remainder comprised additional deletion alleles. **B)** Allele frequency distribution at the *TUBB3* target site in the TUBB3 KD clone. 33% of reads carried a 10 bp deletion, predicted to cause a frameshift, while 66% matched the unmodified reference sequence, consistent with editing of a subset of alleles. Note that the TUBB3 sgRNA designed and used targets the antisense strand, while the sgRNA displayed above is its reverse complement relative to the sense-strand reference.

Supplementary Figure S4: TUBB3 in Mitotic Spindle and Cell Morphology.

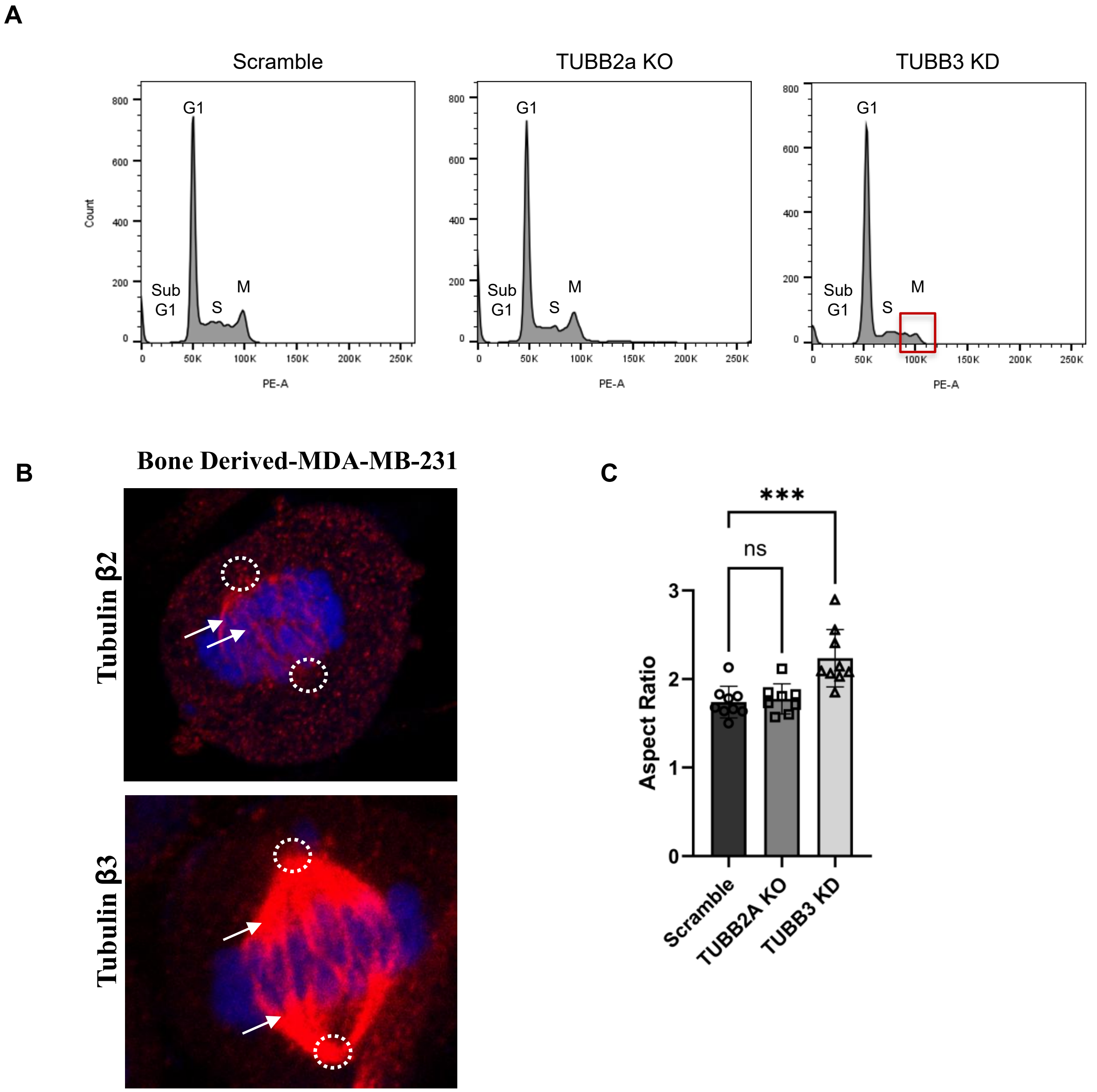

Supplementary Figure S4: TUBB3 in Mitotic Spindle and Cell Morphology. A) Representative flow cytometry graphs, PE-A by Count, corresponding to the analysis shown in Figure 3D. **B)** Immunofluorescent staining of tubulin β2 and tubulin β3 in bone-derived MDA-MB-231 cells. Image depicts strong staining of tubulin β3 on mitotic spindles. The reduction of tubulin β3 may be implicated in spindle assembly defects, leading to inefficient chromosome segregation, reduced mitotic entry, and disrupted cell cycle progression. These defects may contribute to genomic instability and altered proliferative capacity. **C)** Cell aspect ratio (MajorAxisLength/MinorAxisLength) was quantified using CellProfiler. Data is presented as per-image means ± SEM (n=9 images per group) with a total of 930 cells analyzed (Scramble, 384; TUBB2A KO, 298; TUBB3 KD, 248). TUBB3 KD significantly increased cell elongation with an aspect ratio (2.24 ± 0.11) relative to scramble (1.74 ± 0.06). Statistics performed on per-image means via one-way ANOVA with Dunnett's post hoc test versus Scramble (***p < 0.001).

Supplementary Figure S5: Additional Isotype-Specific Compensation

A)

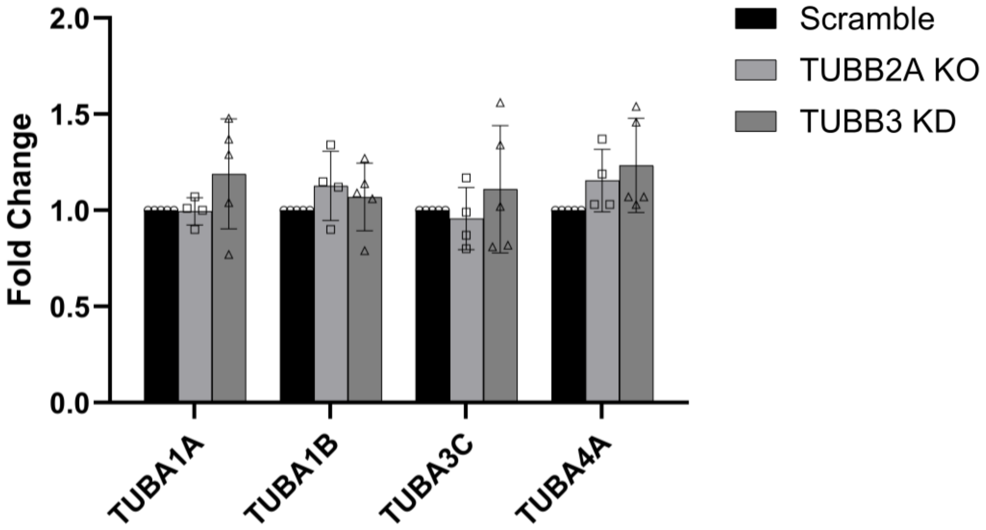

B)

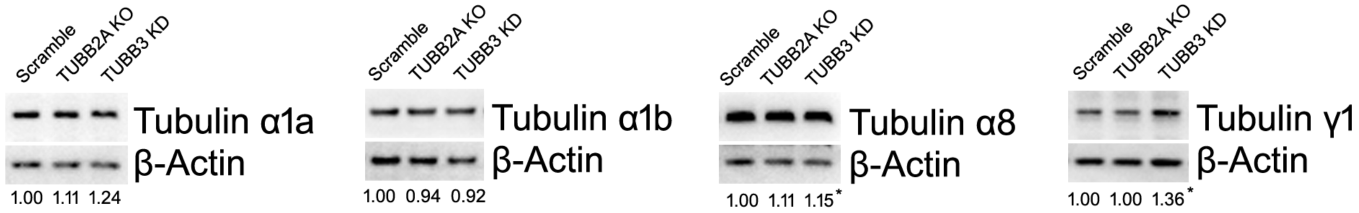

Supplementary Figure S5: Additional Isotype-Specific Compensation. A) Quantitative PCR analysis of α-tubulin isotype expression (*TUBA1A*, *TUBA1B*, *TUBA3C*, *TUBA4A*) in scramble, TUBB2A KO, and TUBB3 KD cells. Expression was calculated using the ΔΔCt method normalized to the average of two housekeeping genes (*ACTB* and *GAPDH*) and is presented as fold change relative to scramble. No significant differences were detected in either TUBB2A KO or TUBB3 KD cells relative to scramble. Bars represent mean ± SD of n=4–5 independent biological samples. Two-way ANOVA with Dunnett's multiple comparisons test. **B)** Western blot analysis of tubulin α1a, α1b, α8, and γ1 protein expression in scramble, TUBB2A KO, and TUBB3 KD cells. TUBB3 KD cells showed increased γ1 (1.36-fold, *$p<0.05$) and a modest increase in α8 (1.15-fold, *$p<0.05$) relative to scramble. No significant changes were observed in TUBB2A KO cells. Quantification represents fold change relative to scramble, with β-actin serving as a loading control. Bars represent mean ± SD of n=4 independent biological samples. One-way ANOVA with Dunnett's multiple comparisons test. Please note that mRNA data was neither obtained for *TUBA8* nor *TUBG1*, and antibodies suitable for α3c and α4a detection were not available.

Supplementary Figure S6: Full Microtubule Regrowth Time Course

A)

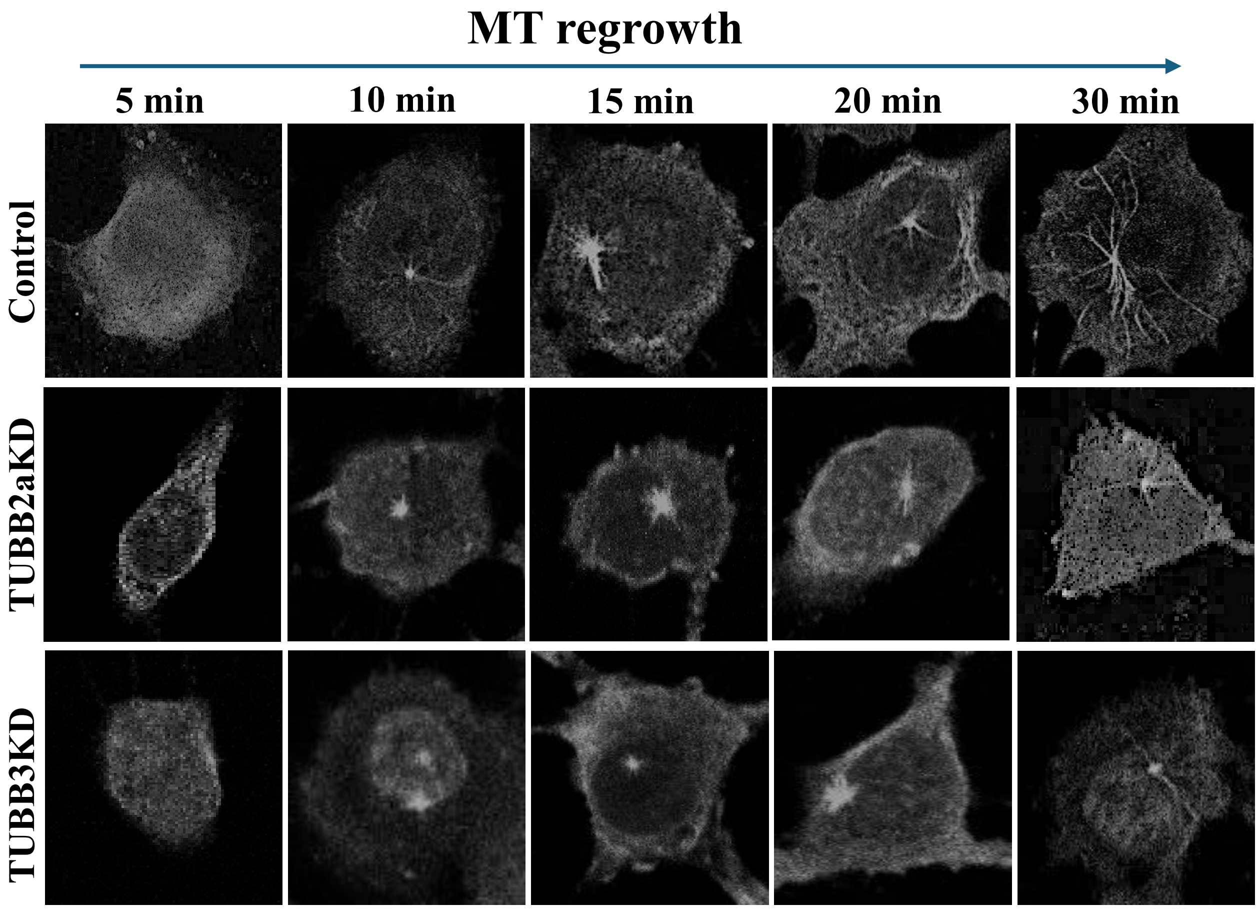

B)

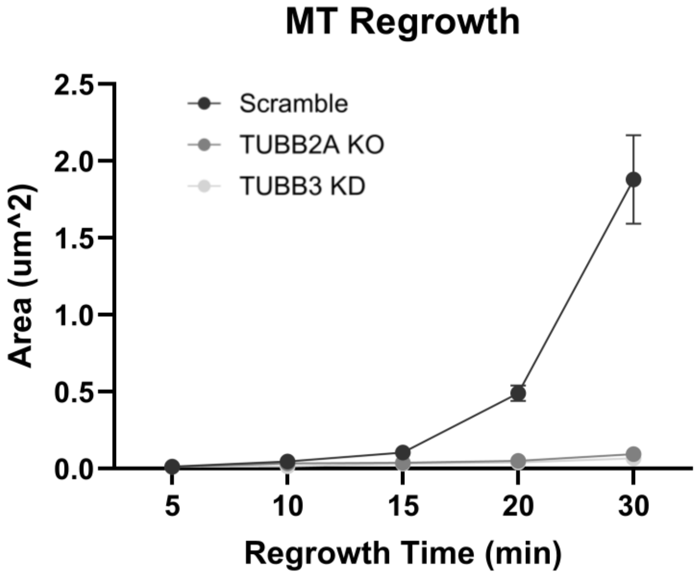

Supplementary Figure S6: Full Microtubule Regrowth Time Course. A) Representative images of scramble, TUBB2A KO, and TUBB3 KD cells at 5-, 10-, 15-, 20-, and 30-min following microtubule recovery. **B)** Quantification of mean aster area per image across the full recovery time course. Aster area was comparable across genotypes at 5-min, indicating intact nucleation. Scramble cells underwent progressive microtubule regrowth, reaching 1.88 μm² by 30 min, whereas both tubulin-deficient lines remained near-baseline throughout (TUBB2A KO, 0.095 μm²; TUBB3 KD, 0.068 μm² at 30 min). Both TUBB2A KO and TUBB3 KD diverge from scramble by 15-min, reaching significance by 20-min. Aster area was measured in a total of 2,105 cells across 125 images (Scramble, 657 cells/41 images; TUBB2A KO, 799/43; TUBB3 KD, 649/41). Per-image means were used as the unit of analysis, with n=5–10 images per genotype per timepoint. Points represent mean± SEM.

Supplementary Figure S7: Binning Rab5-Puncta Size

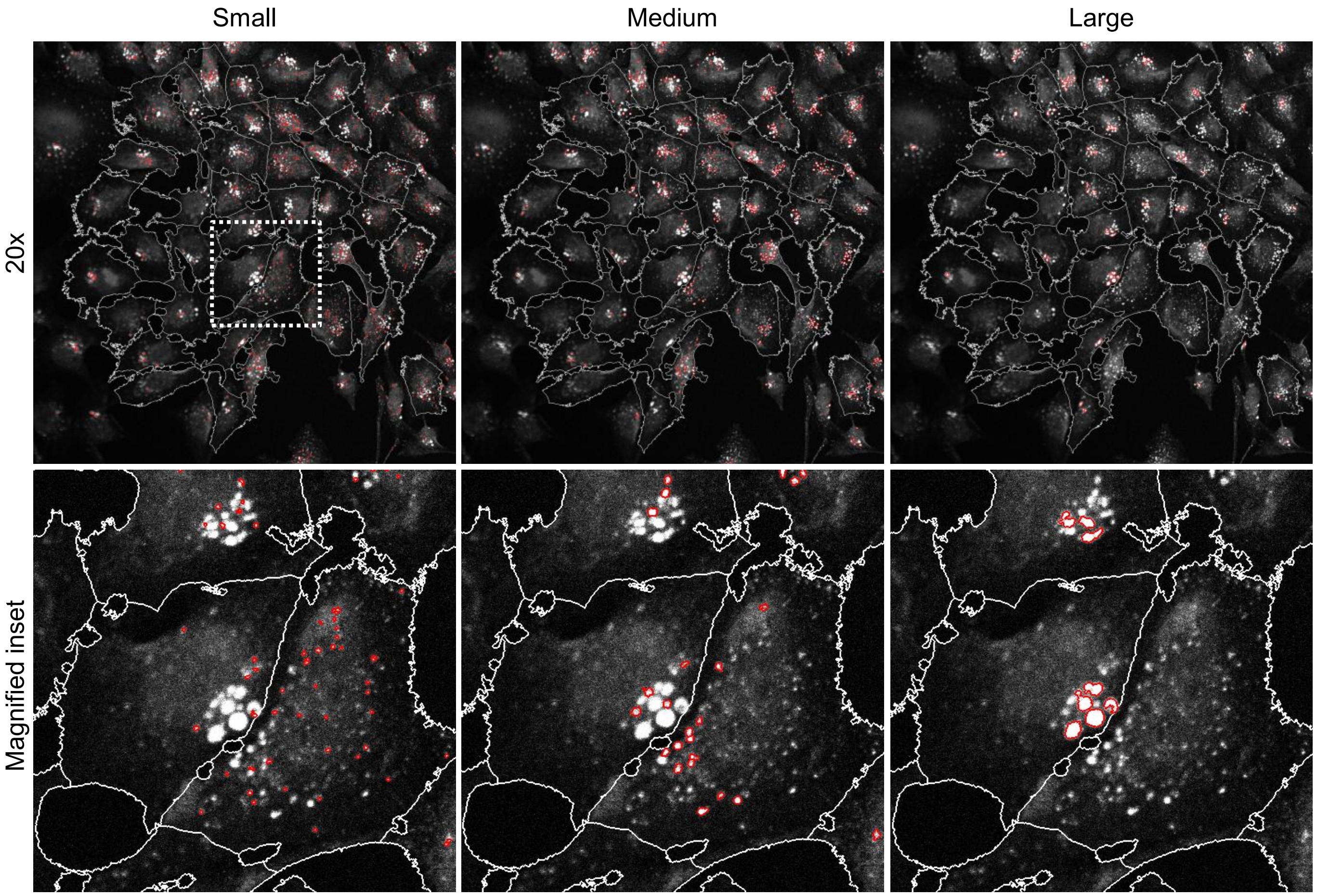

Supplementary Figure S7: Binning Rab5-Puncta Size. Representative immunofluorescence image from TUBB2A KO cells demonstrating Rab5 puncta size quantification. The top row shows a 20x inverted image highlighting Rab5 signal (white) and cell contours (white outline). The bottom row shows a digitally magnified view of the boxed region from the top-left image. Rab5-positive puncta were classified by diameter as small (0.5–1.0 µm), medium (1.0–2.0 µm), or large (2.0–4.0 µm). Red circles depict puncta categorized into their respective groups. Contouring performed via CellProfiler.

Supplementary Figure S8: Gene Ontology Enrichment Analysis of TUBB2A KO and TUBB3 KD cells

A

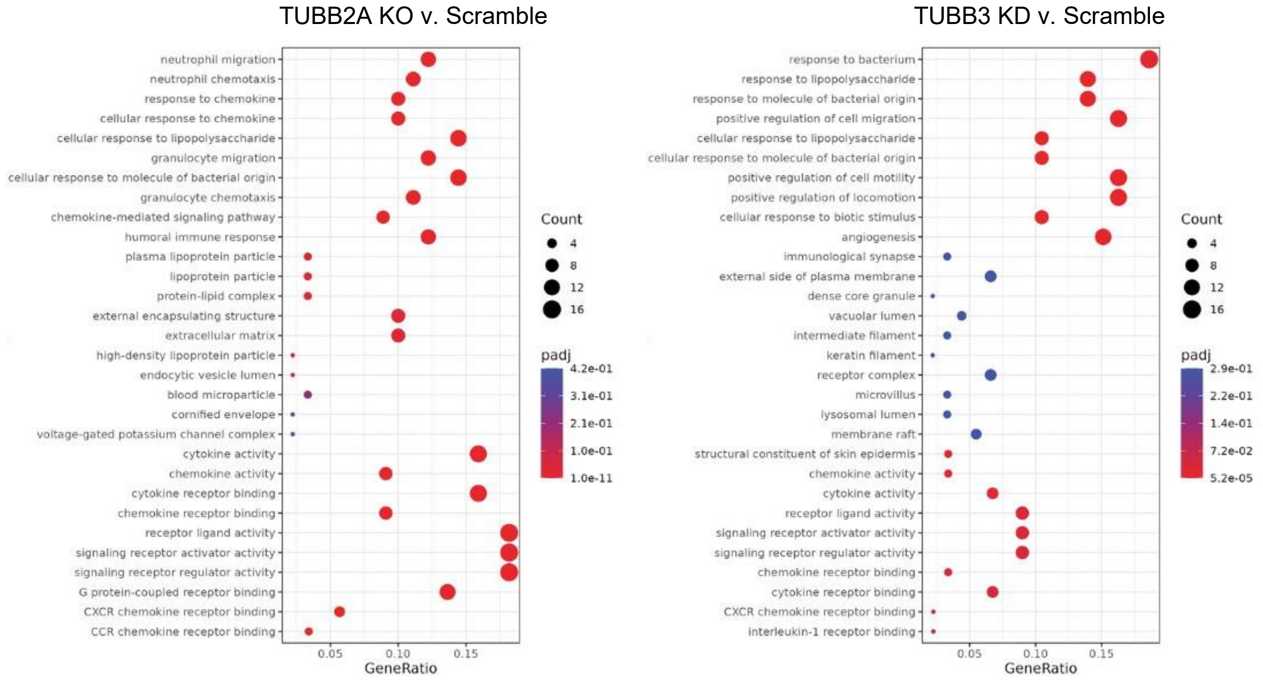

B)

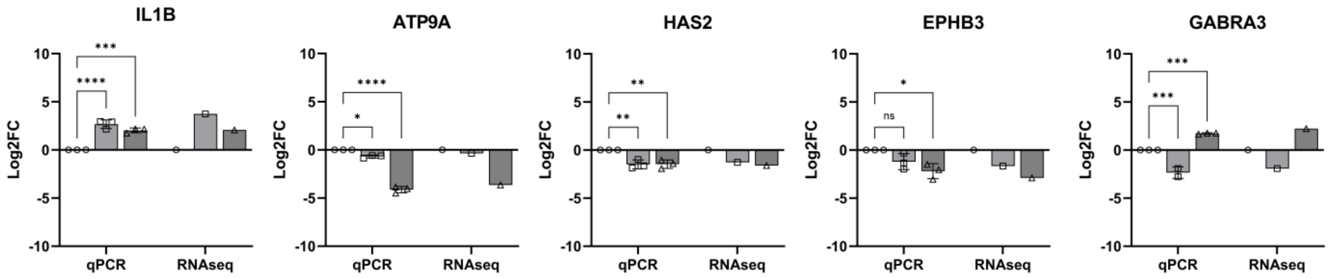

Supplementary Figure S8: Gene Ontology Enrichment Analysis of TUBB2A KO and TUBB3 KD cells. A) Gene ontology (GO) enrichment analysis of the differentially expressed genes identified in Figure 5A for TUBB2A KO (left) and TUBB3 KD (right) cells. Enrichment analysis was performed by Novogene using the complete annotated *Homo sapiens* gene set as background. Terms are ranked by adjusted p-value; dot size represents the number of differentially expressed genes annotated to each term and dot color represents adjusted p-value. **B)** Quantitative PCR validation of representative transcriptional changes identified by RNAseq (IL1B, ATP9A, HAS2, EPHB3, GABRA3). qPCR expression was calculated using the ΔΔCt method normalized to the average of two housekeeping genes (ACTB and GAPDH) and is presented as Log2 fold change relative to scramble control. Each panel pairs qPCR data with the corresponding RNAseq Log2FC for directional comparison. qPCR bars represent mean ± standard deviation from n=3 independent biological samples; statistical significance was assessed by one-way ANOVA with Dunnett's multiple comparison test against scramble control. RNAseq values derive from a single sequencing library per genotype (n=1) and are shown for directional reference only. Significance levels: *$p < 0.05$, **$p < 0.01$, ***$p < 0.001$, ****$p < 0.0001$.

Supplementary Figure S9: Corresponding Brightfield Images of Adhesion Assay

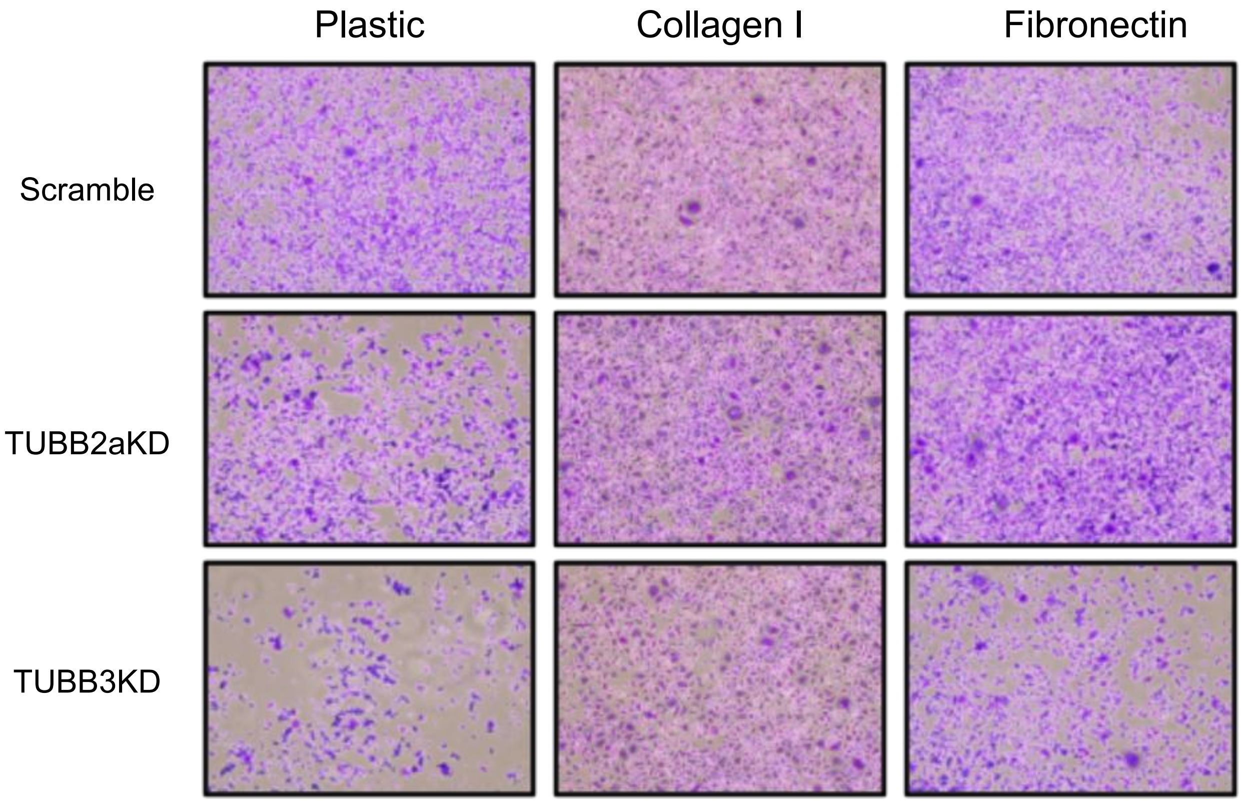

Supplementary Figure S9: Corresponding Brightfield Images of Adhesion Assay. Representative 10x brightfield images of scramble, TUBB2A KO, and TUBB3 KD cells 2 hours after seeding onto uncoated plastic, type I collagen-, or fibronectin-coated plates. TUBB2A KO cells showed increased adhesion to both plastic and fibronectin relative to scramble, whereas TUBB3 KD increased to fibronectin but decreased to plastic. Images are representative of n=4 independent experiments.

Supplemental Figure S10: Focal Adhesion Protein Expression in Tubulin-Deficient Cells

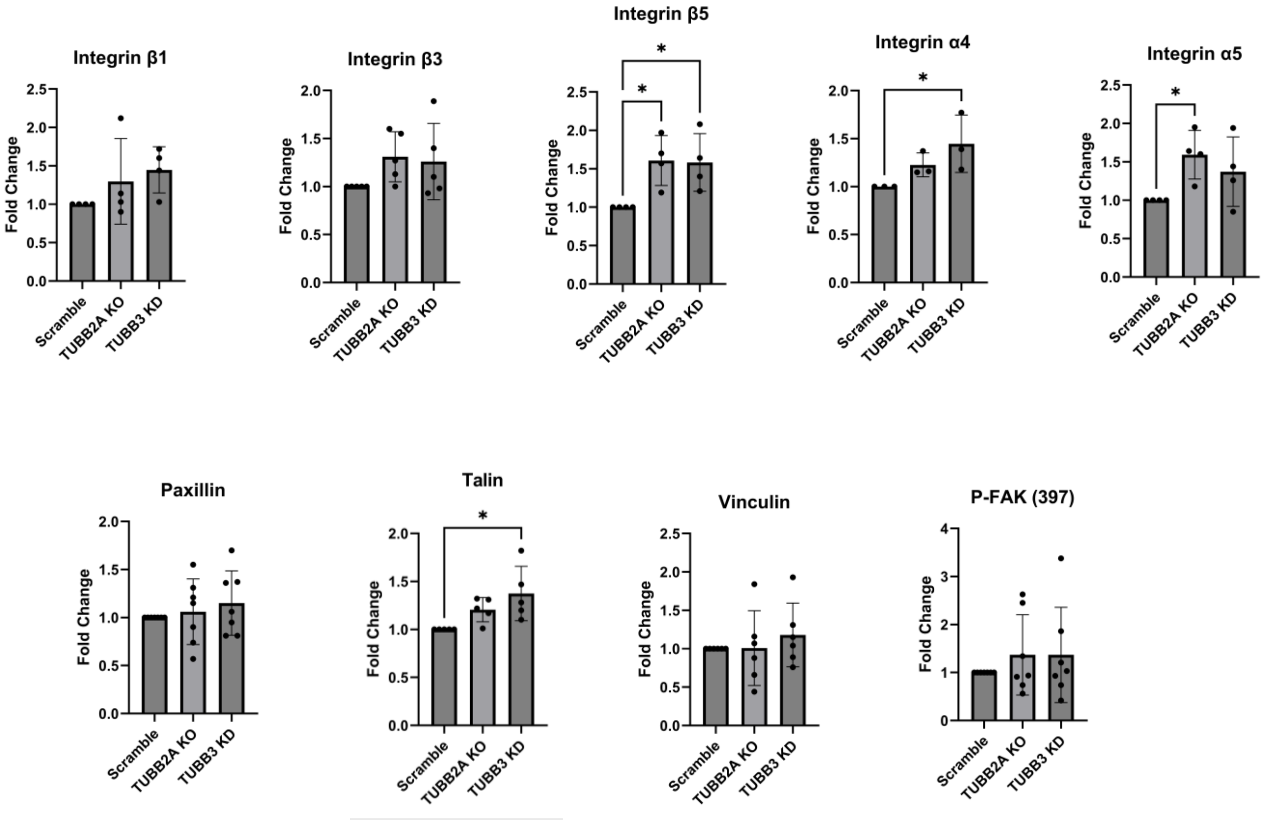

Supplementary Figure S10: Focal Adhesion Protein Expression in Tubulin-Deficient Cells. Bar graphs show average fold change values normalized to scramble control cells. Error bars indicate standard deviation. Sample sizes ranged from n = 3–7 independent sample sets per group. Fold change values correspond to those shown in Figure 7D. Statistical analysis was performed using ordinary one-way ANOVA with Dunnett's multiple comparisons test: $^*p < 0.05$.
